# Comparative Transcriptomics and Proteomics of *Atractylodes lancea* in Response to Endophytic Fungus *Gilmaniella* sp. AL12 Reveals Regulation in Plant Metabolism

**DOI:** 10.3389/fmicb.2019.01208

**Published:** 2019-05-28

**Authors:** Jie Yuan, Wei Zhang, Kai Sun, Meng-Jun Tang, Piao-Xue Chen, Xia Li, Chuan-Chao Dai

**Affiliations:** ^1^Jiangsu Key Laboratory for Microbes and Functional Genomics, Jiangsu Engineering and Technology Research Center for Industrialization of Microbial Resources, College of Life Sciences, Nanjing Normal University, Nanjing, China; ^2^Jiangsu High Quality Rice Research and Development Center, Nanjing Branch of Chinese National Center Rice Improvement, Institute of Food Crops, Jiangsu Academy of Agricultural Sciences, Nanjing, China

**Keywords:** *Atractylodes lancea*, endophytic fungi, beneficial interaction, plant immunity, metabolism, terpenoid biosynthesis

## Abstract

The fungal endophyte *Gilmaniella* sp. AL12 can establish a beneficial association with the medicinal herb *Atractylodes lancea*, and improve plant growth and sesquiterpenoids accumulation, which is termed “double promotion.” Our previous studies have uncovered the underling primary mechanism based on some physiological evidences. However, a global understanding of gene or protein expression regulation in primary and secondary metabolism and related regulatory processes is still lacking. In this study, we employed transcriptomics and proteomics of *Gilmaniella* sp. AL12-inoculated and *Gilmaniella* sp. AL12-free plants to study the impact of endophyte inoculation at the transcriptional and translational levels. The results showed that plant genes involved in plant immunity and signaling were suppressed, similar to the plant response caused by some endophytic fungi and biotroph pathogen. The downregulated plant immunity may contribute to plant-endophyte beneficial interaction. Additionally, genes and proteins related to primary metabolism (carbon fixation, carbohydrate metabolism, and energy metabolism) tended to be upregulated after *Gilmaniella* sp. AL12 inoculation, which was consistent with our previous physiological evidences. And, *Gilmaniella* sp. AL12 upregulated genes involved in terpene skeleton biosynthesis, and upregulated genes annotated as β-farnesene synthase and β-caryophyllene synthase. Based on the above results, we proposed that endophyte-plant associations may improve production (biomass and sesquiterpenoids accumulation) by increasing the source (photosynthesis), expanding the sink (glycolysis and tricarboxylic acid cycle), and enhancing the metabolic flux (sesquiterpenoids biosynthesis pathway) in *A. lancea*. And, this study will help to further clarify plant-endophyte interactions.

## Introduction

Medicinal plants are rich in active compounds such as artemisinin (Sharma and Agrawal, 2013) and ginseng saponin (Wei et al., 2018), which are important sources of modern drugs. Within the increasing population pressure, costs and side effects of drugs, demands for the uses of medicines from plants for treatment of various human ailments are increasing. Compared with synthetic medicines, compounds from plants are thought to be safe to human beings and the ecosystem (Nema et al., 2013). To meet the market demand for these medicinal materials, artificial cultivation technology has been employed for planting medicinal herbs in some Asian countries (Zhou et al., 2005; Sharma et al., 2010; Oh et al., 2014; Lv et al., 2017). However, it is difficult to guarantee the quality and quantity of artificially cultivated medicinal plants because they are more vulnerable to infections by pests and pathogens. Thus, it is imperative to seek effective methods for medicinal plant cultivation. In the past few years, endophytic fungi possessing plant growth promoting properties have been an effective tool for medicinal plant cultivation (Mandal et al., 2013; Ming et al., 2013; Sharma and Agrawal, 2013; Zheng et al., 2016; Zhai et al., 2018).

*Atractylodes lancea* (Thunb.) DC., belonging to the Asteraceae family, is an endangered traditional Chinese medicinal herb (Wang et al., 2008). Its bioactive component, the sesquiterpenoids, possesses various pharmacology properties such as antibacterial, antitumour, and immunomodulation abilities (Wang et al., 2008; Koonrungsesomboon et al., 2014; Na-Bangchang et al., 2017). Over the past few years, natural sources of *A. lancea* have been in short supply because of the excessive exploitation and slow growth rate of the herb (Zhou et al., 2016). The medicinal source of *A. lancea* mainly derives from artificial cultivation, but the yield and quality of this herb are relatively low (Zhou et al., 2016). At present, it is urgent to improve the quality and quantity of the herb as the market demand for *A. lancea* is increasing on a daily basis. The endophytic fungus *Gilmaniella* sp. AL12 isolated from stem of *A. lancea* can establish a beneficial interaction with the host plant (Wang et al., 2012) and promote plant growth and sesquiterpenoid accumulation of tissue culture seedings, which is termed the “double promotion” effect of the endophyte on *A. lancea* (Yuan et al., 2016b). Consistent with this phenomenon, the endophytic fungi AL12 promotes plant growth and sesquiterpenoid accumulation within two years of growth in field experiments. Therefore, a beneficial interaction of *Gilmaniella* sp. AL12 with *A. lancea* is considered suitable for cultivation of *A. lancea* and will provide a theoretical reference for endophytic fungi-medicinal herb interactions.

In view of the limited carbon and energy source in plants, the accumulation of secondary metabolites occurs at the cost of primary metabolism, representing a discrepancy with the “double promotion” effect of *Gilmaniella* sp. AL12 on *A. lancea*. The plant growth promotion effect of the endophyte on *A. lancea* has been preliminarily ascribed to nutrient assimilation, photosynthesis, and phytohormone content regulation (Yuan et al., 2016b). Moreover, the enhanced sesquiterpenoids accumulation of *A. lancea* has been shown to be mediated by multiple defense related signals of the host induced by the endophyte (Wang et al., 2011; Ren and Dai, 2012, 2013; Yuan et al., 2016a). Given that primary metabolism-dependent terpenoid precursor biosynthesis and secondary metabolism-related terpenoid skeleton biosynthesis and transformation are simultaneously involved in sesquiterpenoid synthesis (Dudareva et al., 2006; Chen W. et al., 2017; Sharma et al., 2017; Vattekkatte et al., 2018), the molecular and biochemical regulation of the plants relevant to primary and secondary metabolism should be considered. However, thus far, a global understanding of the -regulated expression of genes or proteins in primary and secondary metabolism and related regulatory processes is still lacking.

In this study, we employed transcriptomics and proteomics on endophyte-inoculated and endophyte free plants to better understand the impact of *Gilmaniella* sp. AL12 on plant metabolism and related regulatory processes of *A. lancea* at the transcriptional and translational level. The following four essential questions were addressed in this study: (1) Which plant metabolic or regulatory processes of *A. lancea* are affected by *Gilmaniella* sp. AL12? (2) What is the effect of the fungal endophyte on the regulation of primary metabolism-dependent terpenoid precursor biosynthesis in *A. lancea*? (3) What is the effect of the fungal endophyte on the regulation of secondary metabolism-related terpenoid skeleton biosynthesis and transformation in *A. lancea*? (4) Are other potential signals involved in the fungal endophyte-induced sesquiterpenoids biosynthesis of *A. lancea*?

## Materials and Methods

### Plantlet Material and Fungal Inoculation

*Atractylodes lancea* meristem cultures were established using sterilized plantlets according to our previous studies (Wang et al., 2012). Firstly, meristem cultures were established using mature *A. lancea* planted in Maoshan, Jiangsu Province, China (Wang et al., 2012). Sterile adventitious buds (approximately 2–3 cm long) of young stems were collected and carefully washed under running tap water. They were surface sterilized by immersing in ethanol (75%) for 30 s, followed by soaking in mercury chloride solution (1%) for 10 min and rinsing in sterile distilled water five times (Wang et al., 2012). Subsequent procedures were conducted aseptically (Wang et al., 2012). The explants were transferred in 50 mL Murashige and Skoog medium containing sucrose (30 g L^−1^), agar (10 g L^−1^), naphthaleneacetic acid (0.3 mg L^−1^), and 6-benzyladenine (2.0 mg L^−1^) in 150-mL sealed Erlenmeyer flask to emerge adventitious buds for 4 weeks (Wang et al., 2012). Then, newborn adventitious buds were separated and grown in 50 mL Murashige and Skoog medium containing sucrose (30 g L^−1^), agar (10 g L^−1^), naphthaleneacetic acid (0.3 mg L^−1^) and 6-benzyladenine (2.0 mg L^−1^) in 150-mL sealed Erlenmeyer flask for further differentiation until newborn adventitious buds were suficient for transplantation. After separation, newborn axillary buds were transplanted into 50 mL Murashige and Skoog medium containing sucrose (30 g L^−1^), agar (10 g L^−1^), and naphthaleneacetic acid (0.25 mg L^−1^) in a 150-mL sealed Erlenmeyer flask to develop into rooting plantlets. Each bud was cultured in an Erlenmeyer flask. All plantlets were grown in growth chamber (PGX-600A-12H, Ningbo Lifewww Technology Co., China) with a photoperiod of 12 h, a light density of 3,400 lm m^−2^, and a temperature cycle of 25/18°C day/night. Thirty-day-old rooting plantlets were chosen for the endophytic fungus inoculation experiments. The endophytic fungus *Gilmaniella* sp. AL12 isolated from the stem of *A. lancea* was grown on potato dextrose agar medium (Chen et al., 2008; Wang et al., 2012). After five days of culture at 28°C, 5-mm *Gilmaniella* sp. AL12 mycelial disks were placed near the plant caudexes on the medium for inoculation. Additionally, equal-sized potato dextrose agar disks were used as the control (Yuan et al., 2016a). All plantlets were grown in growth chamber and performed in triplicate.

### Plant Growth and Sesquiterpenoid Content Analysis

Plantlets were harvested at 15 DPI for the shoot dry weight and sesquiterpenoid content analysis. Briefly, harvested plants were dried at 30°C until the weight was constant, and then 1 g of the ground powder was extracted with 4 mL cyclohexane at room temperature for 10 h. After 15 min of sonication (60 Hz), the mixture was centrifuged for 5 min at 5,000 *g* at 4°C. After filtering through 0.22-μm-diameter microporous membranes, the total cyclohexane extract was dried over anhydrous sodium sulfate and stored in a dark glass bottle at 4°C before gas chromatography (GC) analysis (Yuan et al., 2016b).

A GC system (Agilent 7890A, United States) equipped with a fame ionization detector was used for sesquiterpenoid analysis (Yuan et al., 2016b). The GC column was DB-1ms (30 m × 0.32 mm × 0.10 μm) with high-purity nitrogen as a carrier gas at a flow rate of 0.8 mL min^−1^. For sesquiterpenoid analysis, 1 μl of cyclohexane was directly injected onto the GC column with the following temperature program: injection at 240°C, an initial temperature of 100°C (4 min hold) raised to 140°C at a rate of 10°C min^−1^ (10-min hold), followed by an increase to 220°C at a rate of 10°C min^−1^ (10-min hold), raised to 260°C at a rate of 10°C min^−1^ (2-min hold). The detector temperature was then set at 350°C. As previously described, qualitative and quantitative analyses of seven sesquiterpenoids were performed according to authentic standards (Yuan et al., 2016b).

### Determination of Photosynthetic Parameters

Plantlets were harvested at 10: 00 AM at 15 DPI for Chlorophyll a fluorescence measurement using a Handy-PEA instrument (Handy PEA, Hansatech, United Kingdom) (Wang et al., 2017). Prior to measurement, *A. lances* leaves were dark-adapted for 30 min at room temperature to relax the reaction centers. The leaves were exposed to red light of 650 nm at an excitation irradiance of 3,000 μmol m^−2^ s^−1^ for 800 ms. Then, fluorescence parameters were calculated according to Supplementary Table S1.

### RNA Sequencing and Functional Annotation of Differentially Expressed Genes

Plantlets were harvested at 15 DPI. Shoot tissue of endophyte-inoculated or endophyte-free plants were harvested for RNA extraction using TRIzol reagent (Invitrogen, CA, United States) (Chen F. et al., 2017). After quality and purity checking, equal amounts of RNA samples from three biological replicates of CK (endophyte-free plants for control) and AL12 (endophyte-inoculated plants) were used for construction of six mRNA-Seq libraries. Libraries were indexed, pooled and then sequenced on an Illumina HiSeq^TM^ 4000 sequencing platform (Vazyme Biotech Co., Nanjing, China). After filtering, the remaining high-quality reads were assembled *de novo* by using the Trinity program. Certain short reads with overlapping regions were assembled into longer clusters (contigs). Paired-end reads were used to fill scaffolds gaps to obtain unigenes. Based on a BLASTX search (E value < 10^−5^), functional annotation of these unigenes was performed with the following public databases, including the National Center for Biotechnology Information (NCBI) non-redundant protein (NR) and nucleotide (NT) database, Swiss-Prot protein database, Gene Ontology (GO) database, Cluster of Orthologous Groups (COGs) database, and Kyoto Encyclopedia of Genes and Genomes (KEGG) database. The fragments per kilobase of transcript per million mapped reads (FPKM) method was used to determine the unigene expression. Differentially expressed genes (DEGs) between CK and AL12 samples were identified with log_10_ (AL12_FPKM_/CK_FPKM_) ≥ 1 and a false discovery rate (FDR) ≤ 0.05. Subsequently, upregulated and downregulated DEGs were conducted by GO and KEGG enrichment analysis, respectively. The RNA sequencing data for this article were submitted to the NCBI Sequence Read Archive (SRA) under accession number SRP132616.

### Real-Time Quantitative PCR Validation

Real-time quantitative PCR (RT-qPCR) was performed to determine the expression levels of differentially expressed genes of *A. lancea* cultured *in vitro*. Elongation factor 1 alpha gene (*EF1a*) was used as an internal reference (Yuan et al., 2016b). Primers for the selected DEGs are listed in Supplementary Table S2. One microgram of total RNA was transcribed into cDNA using HisCRIPT^^®^^ II Q RT SuperMix for qPCR (+gDNA wiper) (Vazyme Biotech Co., Nanjing, China) according to the manufacturer’s instruction. Subsequently, RT-qPCR was conducted using the DNA Engine Opticon 2 Real-time PCR Detection System (Bio-Rad, Hercules, CA, United States). The reaction system consisted of a volume of 20 μL, which included 10 μL of 2 × AceQ qPCR SYBR^^®^^ Green Master Mix (High ROX Premixed) (Vazyme Biotech Co., Nanjing, China), 2 μL of the cDNA template, 0.4 μL of each primer, and 7.6 μL ddH_2_O. The reaction conditions were 95°C for 5 min, followed by 40 cycles of 95°C for 30 s, 60°C for 30 s, 95°C for 15 s, 60°C for 60 s, and 95°C for 15 s. All assays were performed in triplicate. Relative expression levels for each cDNA sample were calculated by the 2^−ΔΔCt^ method (Wang et al., 2015; Liu et al., 2018).

### Total Protein Extraction, Two-Dimensional Gel Electrophoresis (2-DE) Separation, Image Analysis, In-Gel Digestion, Protein Identification and Functional Analysis

Plant shoots were harvested at 15 DPI for protein extraction. Protein was extracted using the trichloroacetic acid/acetone precipitation method (Wang et al., 2016). For this purpose, 2 g of shoot material was frozen in liquid nitrogen, ground into a fine powder, mixed with 10 mL cold trichloroacetic acid/acetone buffer (13% (w/v) trichloroacetic acid, 0.07% (v/v) 2-mercaptoethanol in acetone, and kept overnight at −20°C. After shaking for 1 h, the proteins were separated by centrifugation (10,000 *g*, 15 min, 4°C), and washed twice with cold acetone and once with 80% (v/v) acetone. The proteins were then air-dried and dissolved in protein lysis buffer (7 M urea, 2 M thiourea, 4% (w/v) 3-[(3-cholamidopropyl) dimethylam-monio]-1-propanesulphonate (CHAPS), 2% (v/v) IPG buffer, 1% (v/v) phenylmethanesulphonyl fluoride (PMSF), 10 mM Na_3_VO_4_, 10 mM NaF, 50 mM glycerophosphate, 5 μg mL^−1^ antiprotease, 5 μg mL^−1^ trasylol and 5 μg mL^−1^ leupeptin). After sonication and centrifugation, the resulting protein extraction was quantified using a commercial dye reagent (Bio-Rad Laboratories, Hercules, CA, United States).

Proteins (900 μg) were separated using immobilized pH gradient (IPG) strips (pH 3–10, nonlinear gradient, 24 cm) and rehydrated for 12 h. First-dimension isoelectric focusing was performed at 20°C for 1 h at 250 V, 2 h at 1,000 V, 5 h at 10,000 V, and 12 h at 500 V (Chen et al., 2016). The strips were then equilibrated with a buffer containing 6 M urea, 2% (w/v) sodium dodecyl sulphate (SDS), 75 mM Tris-HCl (pH 8.8), 30% (v/v) glycerol, and 1% (w/v) dithiothreitol (DTT). In the second dimension, 12% (w/v) sodium dodecyl sulfate polyacrylamide gel electrophoresis (SDS-PAGE) was used, and then coomassie brilliant blue stained (GE Healthcare). Gel images were scanned using an Image Scanner III (GE Healthcare) and analyzed using the Image Mater 2D Platinum 7.0 (GE Healthcare). Each spot was normalized to a relative volume. Quantitative analysis sets were created between each control and AL12-inoculated plants, and each treatment was performed in triplicate.

Differentially expressed spots were selected, manually excised, digested with trypsin (Tatli et al., 2017), and analyzed by UltrafleXtreme TOF/TOF (Bruker Daltonics, Germany). Reflector positive mode was used, with a wavelength of 355 nm and acceleration voltage of 2 kV. MS and MS/MS data were analyzed, and peak lists were generated using FlexAnalysis 3.1 (Bruker Daltonics, Germany). MS and MS/MS analyses were compared to the NCBI green plant protein database using the MASCOT 2.4 search engine (Matrix Science, London, United Kingdom) (Chen et al., 2016; Tatli et al., 2017). Search parameters were as follows: trypsin digestion with one missed cleavage, variable modifications (oxidation of methionine and carbamidomethylation of cysteine), and mass tolerance of a precursor ion and fragment ion at 0.2 Da for +1 charged ions. For all proteins successfully identified by a Peptide Mass Fingerprint and/or MS/MS, a Mascot score greater than 50 (the default MASCOT threshold for such searches) was accepted as significant (*P* < 0.05) (Tatli et al., 2017).

### Statistical Analysis

The mean values and standard deviations were calculated using SPSS Statistics 17.0 software (SPSS Inc., Chicago, United States), and the statistical evaluation between two treatments was compared using the independent-samples *t*-test. Values are the means of three independent experiments. Bars represent standard deviations. Asterisks denote significant differences from the control (*t*-test; ^∗^*P* < 0.05; ^∗∗^*P* < 0.01).

## Results

### *Gilmaniella* sp. AL12 Improved Shoot Growth and Sesquiterpenoid Accumulation in *A. lancea*

**FIGURE 1:**
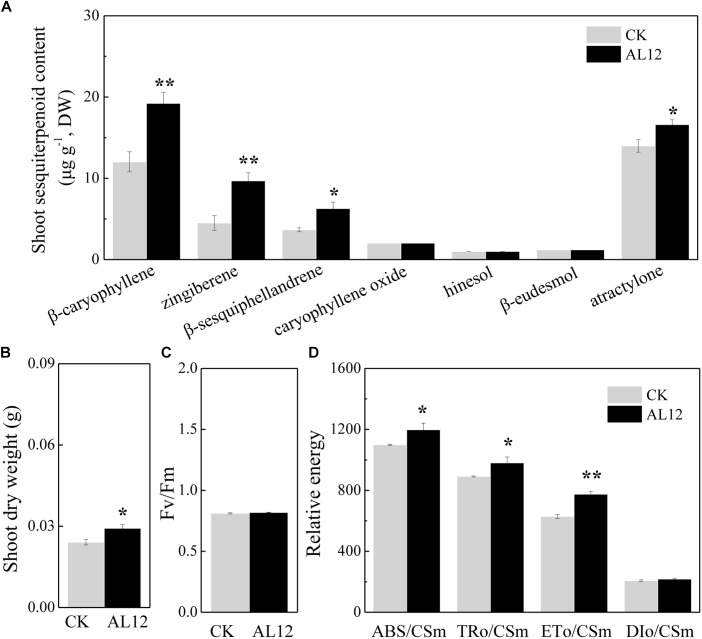
The effect of the fungal endophyte *Gilmaniella* sp. AL12 inoculation on shoot sesquiterpenoid content **(A)**, shoot dry weight **(B)**, chlorophyll fluorescence relative value **(C)**, and phenomenological fluxes per cross section **(D)** of *A. lancea*. Thirty-day-old plantlets treated with 5-mm AL12 mycelial disks were harvested at 15 DPI. Controls were established using equal sized potato dextrose agar disks. Values are the means of three independent experiments. Bars represent standard deviations. Asterisks denote significant differences from the control (*t*-test; ^∗^*P* < 0.05; ^∗∗^*P* < 0.01).

The content of β-caryophyllene, zingiberene, β-sesquiphellandrene, caryophyllene oxide, and atractylone in shoots of *A. lancea* all significantly increased after fungal endophyte inoculation (Figure 1A). The shoot dry weight of endophyte-inoculated plants was higher than that of endophyte-free *A. lancea* (Figure 1B). And, we analyzed chlorophyll fluorescence parameter of endophyte-inoculated and endophyte-free *A. lancea* (Figure 1C,D). The active photosynthesis II (PSII) reaction center captures of light energy to transform into excitation energy, then converts part of excitation energy into chemical energy, thus promotes carbon assimilation reaction, and the rest of excitation energy dissipates (Li et al., 2005). Although the maximum quantum yield of primary photochemistry (F_v_/F_m_) of *A. lancea* was unchanged after endophyte inoculation (Figure 1C), the endophyte increased phenomenological fluxes per cross section including absorption flux of photons (ABS/CS_m_), phenomenological fluxes for trapping (TR_o_/CS_m_), and potential electron transport (ET_o_/CS_m_) of *A. lancea* (Figure 1D), indicating that the fungal endophyte improved PS II reaction center performance in *A. lancea.*

### *Gilmaniella* sp. AL12 Induced Shoot Transcriptional Changes in *A. lancea*

**FIGURE 2:**
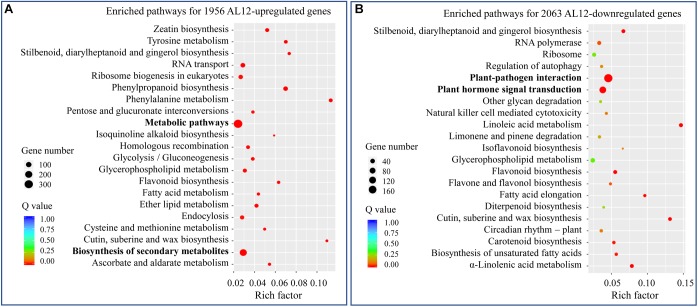
KEGG analysis of differentially expressed genes in shoots of *A. lancea* after the fungal endophyte *Gilmaniella* sp. AL12 inoculation. **(A)** The top 21 enriched KEGG pathways of AL12 upregulated and **(B)** AL12 downregulated genes in shoots of *A. lancea*.

We analyzed the transcriptome of *A. lancea* shoots inoculated or not inoculated with endophytic fungi AL12. A total of 1956 upregulated and 2063 downregulated genes were identified (Supplementary Figure S1). To further elaborate the function of the DEGs, we first conducted GO enrichment analysis (Supplementary Figure S2). The most upregulated DEGs belonged to oxidation-reduction-related GO terms. Among the downregulated DEGs, stress response-related GO terms were the most enriched. We further performed KEGG pathway enrichment analysis for the upregulated and downregulated DEGs (Figure 2). For the upregulated DEGs, “metabolic pathway” and “biosynthesis of secondary metabolism” were the most enriched KEGG pathways (Figure 2A), while “plant-pathogen interaction” and “plant hormone signal transduction” were significantly enriched pathways for the downregulated DEGs (Figure 2B).

The DEGs were functionally related to known metabolic pathways, secondary metabolic pathways, and regulatory processes using the online software iPath2: interactive Pathways Explorer^1^. And, pathways including transcription, translation, replication and repair, protein folding, and transport tend to be upregulated, while protein degradation pathway tend to be downregulated in shoots of *A. lancea* after the fungal endophyte inoculation (Supplementary Table S3). Compared with non-inoculated plants, in endophyte-inoculated plants, nucleotide metabolism, glycan biosynthesis and metabolism, and lipid metabolism pathways tends to be downregulated (Supplementary Table S4). Additionally, the endophytic fungus AL12 upregulated the energy, carbohydrate, amino acid, cofactor and vitamin, and secondary metabolism pathways in shoots of *A. lancea* (Supplementary Tables S5–S7).

### *Gilmaniella* sp. AL12 Upregulated Genes Involved in Primary Metabolism

**FIGURE 3:**
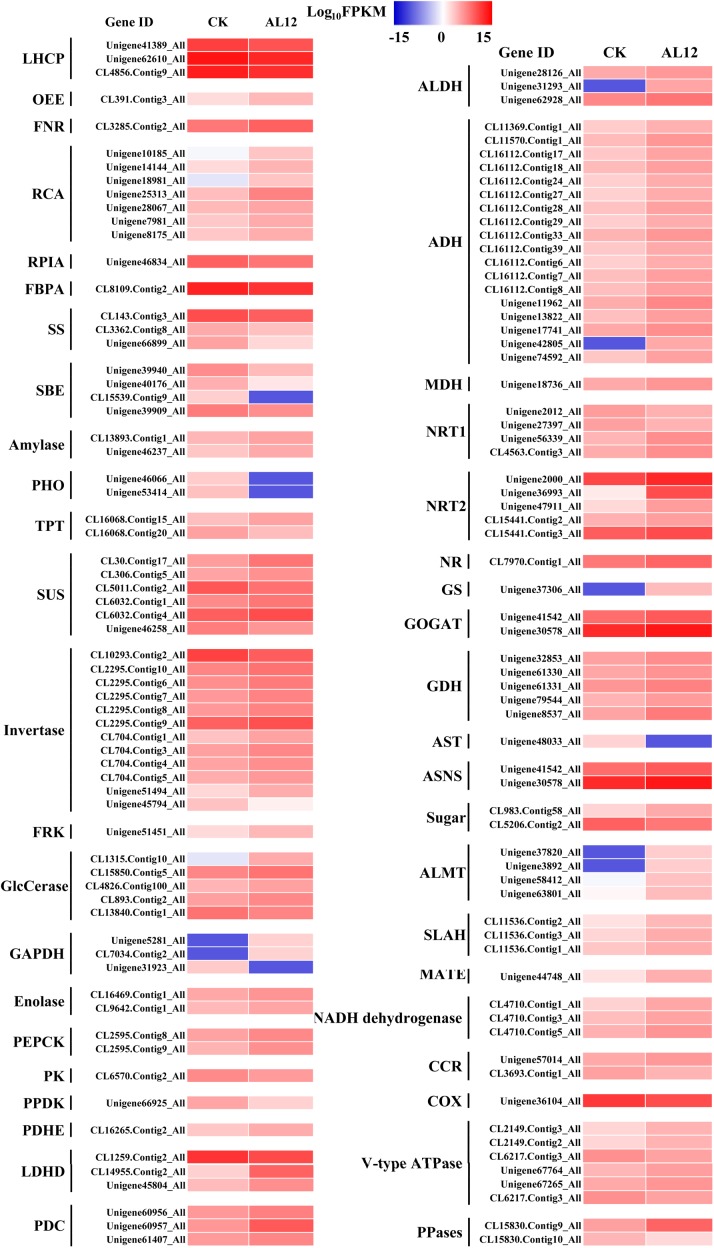
Differentially expression genes involved in photosynthesis, carbon/nitrogen metabolism, and oxidative phosphorylation in shoots of *A. lancea* after the fungal endophyte *Gilmaniella* sp. AL12 inoculation. LHCP, light-harvesting chlorophyll a/b-binding protein; OEE, PSII oxygen-evolving enhancer protein; FNR, Ferredoxin-NADP reductase; RCA, ribulose bisphosphate carboxylase/oxygenase activase; RPLA, Ribose 5-phosphate isomerase; FBPA, fructose-bisphosphate aldolase; SS, starch synthase; SBE, 1,4-alpha-glucan branching enzyme; amylase, PHO, starch phosphorylase; TPT, Glucose-6-phosphate/phosphate-translocator; SUS, sucrose synthase; invertase, β-fructosidases; FRK, fructokinase; GlcCerase, Beta-glucosidase-related glycosidases; GAPDH, glyceraldehyde 3-phosphate dehydrogenase; PEPCK, phosphoenolpyruvate carboxykinase (ATP); PK, pyruvate kinase; PPDK, Pyruvate orthophosphate dikinase; PDHE, pyruvate dehydrogenase; LDHD, lactate dehydrogenase; PDC, pyruvate decarboxylase; ALDH, aldehyde dehydrogenase (NAD^+^); ADH, alcohol dehydrogenase; MDH, malate dehydrogenase; NRT1, low-affinity nitrate transporter; NRT2, high-affinity nitrate transporter; NR, nitrate reductase; GS, glutamine synthetase; GOGAT, glutamate synthase; GDH, glutamate dehydrogenase; AST, asparagine transaminase; ASNS, asparagine synthetase; sugar, bidirectional sugar transporter; ALMT, aluminum-activated malate transporter; SLAH, S-type anion channel; MATE, MATE efflux family protein; CCR, cytochrome-c reductase; COX, Cytochrome c oxidase; PPases, Inorganic pyrophosphatase.

Our results revealed that most DEGs annotated as functioning in photosynthesis and oxidative phosphorylation were globally upregulated after AL12 inoculation (Supplementary Table S5 and Figure 3), including genes annotated as ferredoxin-NADP^+^ reductase (FNR), photosystem II oxygen-evolving enhancer protein 2 (OEE), ribulose-1,5-bisphosphate carboxylase/oxygenase activase (RCA), NADH dehydrogenase, and V-type H^+^-transporting ATPase, among others. However, three genes encoding chlorophyll A/B binding protein (LHCP) were down-regulated.

Endophyte inoculation enhanced plant carbon metabolism, with an upregulation of genes involved in carbohydrate metabolism and transport (Figure 3). For instance, most DEGs annotated as phosphate transporter (TPT), amylase, invertase, β-glucosidase (GlcCerase), fructokinase (FRK), glyceraldehyde 3-phosphate dehydrogenase (GAPDH), phosphopyruvate hydratase (enolase), and phosphoenolpyruvate carboxykinase (PEPCK) were upregulated. Interestingly, genes that function in controlling pyruvate and acetyl-CoA biosynthesis were upregulated. For example, AL12 upregulated genes encoding lactate dehydrogenase (LDHD), pyruvate decarboxylase (PDC), and aldehyde dehydrogenase (ALDH), suggesting that it promotes the conversion of lactate into pyruvate and the biosynthesis of acetic acid. Similarly, the expression of genes encoding alcohol dehydrogenase (ADH) and malate dehydrogenase (MDH) was increased after AL12 inoculation, indicating that oxaloacetate (OAA) and acetic acid biosynthesis were improved. Moreover, the endophytic fungus *Gilmaniella* sp. AL12 upregulated genes encoding MDH and pyruvate dehydrogenase (PDHE), thus contributing to acetyl-CoA biosynthesis and OAA regeneration. Consistent with the improved tricarboxylic acid cycle (TCA cycle), seven genes annotated as malate transporter were up-regulated.

The balance of carbon and nitrogen metabolism is very important for plant metabolism. Most DEGs annotated as functioning in nitrogen metabolism were upregulated after AL12 inoculation (Figure 3). These included genes encoding high affinity nitrate transporter (NRT2), glutamate dehydrogenase (GDH), and others. In particular, one gene encoding glutamine synthetase (GS) exhibited a 10-fold increase in expression in response AL12 inoculation. Additionally, most DEGs annotated as functioning in amino acid metabolism were upregulated (Supplementary Table S6).

Most DEGs annotated as functioning in lipid metabolism were downregulated after AL12 treatment (Supplementary Table S4). These DEGs compass fatty acid elongation, the biosynthesis of unsaturated fatty acids, wax biosynthesis, linoleic acid metabolism, alpha-linolenic acid metabolism, and others. In contrast, most genes involved in fatty acid metabolism, steroid biosynthesis, glycerophospholipid metabolism, ether lipid metabolism, and sphingolipid metabolism were upregulated. Among the upregulated genes, it is worth noting that 32 DEGs encoding phospholipase D (PLD) were upregulated after AL12 inoculation, and eight showed a greater than 10-fold increase in expression (Supplementary Table S8).

### *Gilmaniella* sp. AL12 Upregulated Genes Involved in Secondary Metabolism

**FIGURE 4:**
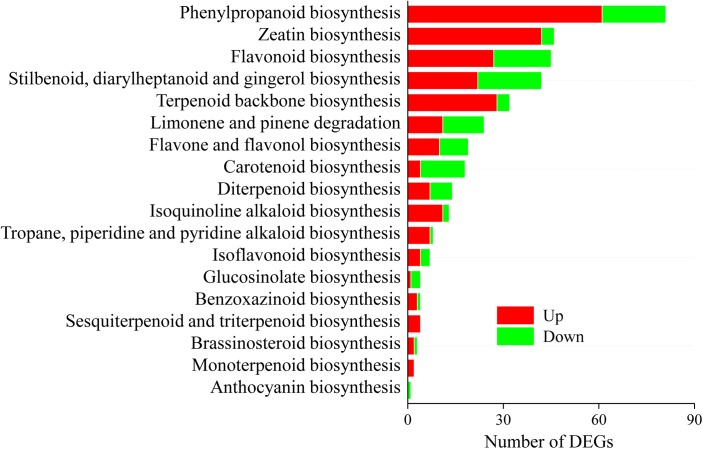
Distribution of differentially expression genes involved in secondary metabolism in shoots of *A. lancea* after the fungal endophyte *Gilmaniella* sp. AL12 inoculation. The *x* axis indicates the KEGG pathway. The *y* axis presents the number of DEGs. Numbers in red block or green blocks represent the number of upregulated or downregulated DEGs, respectively.

A total of 299 DEGs involved in 18 biosynthesis pathways of secondary metabolites were regulated in *A. lancea* after AL12 inoculation (Figure 4). Among these pathways, DEGs involved in eight biosynthesis pathways of secondary metabolites were significantly regulated in *A. lancea* after AL12 inoculation (Supplementary Table S9). The eight pathways include Phenylpropanoid biosynthesis, Zeatin biosynthesis, Flavonoid biosynthesis, Stilbenoid, diarylheptanoid and gingerol biosynthesis, Limonene and pinene degradation, Flavone and flavonol biosynthesis, Carotenoid biosynthesis, and Diterpenoid biosynthesis. The largest subcategory was phenylpropanoid biosynthesis, followed by zeatin biosynthesis. The upregulated DEGs involved in phenylpropanoid biosynthesis were associated with lignin biosynthesis. Additionally, most of the DEGs involved in zeatin biosynthesis were upregulated, particularly adenylate dimethylallyl transferase (IPT). Consistent with the increased sesquiterpenoids content in *A. lance*a shoots (Figure 1A), the expression of genes associated with terpenoid backbone biosynthesis and sesquiterpene biosynthesis was increased (Figure 4 and Supplementary Figure S3). Genes encoding farnesyl diphosphate synthase (FDPS) and β-farnesene synthase were increased after AL12 treatment (Supplementary Figures S3, S4).

### *Gilmaniella* sp. AL12 Downregulated Genes Involved in Plant-Pathogen Interactions

**FIGURE 5:**
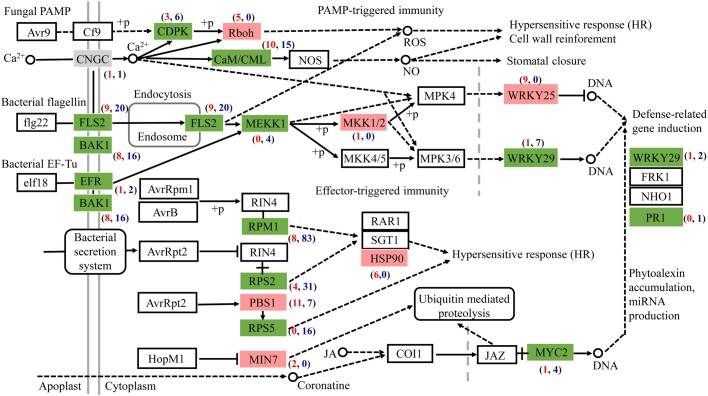
The fungal endophyte *Gilmaniella* sp. AL12 affects the expression of genes involved in plant-pathogen interactions in shoots of *A. lancea*. Red boxes indicate most upregulated genes. Green boxes indicate most downregulated genes. Gray boxes indicate equally upregulated and downregulated genes. CNGC, cyclic nucleotide gated channel; FLS2, LRR receptor-like serine/threonine-protein kinase FLS2; BAK1, brassinosteroid insensitive 1-associated receptor kinase 1; EFR, LRR receptor-like serine/threonine-protein kinase EFR; CDPK, calcium-dependent protein kinase; Rboh, respiratory burst oxidase; CaM/CML, calmodulin/calcium-binding protein CML MEKK1, mitogen-activated protein kinase kinase kinase 1; MKK1/2, mitogen-activated protein kinase kinase 1; WRKY 25, transcription factor WRKY25; WRKY 29, transcription factor WRKY29; RPM1, disease resistance protein RPM1; RPS2, disease resistance protein RPS2; RPS5, disease resistance protein RPS5; PBS1, serine/threonine-protein kinase; MIN7, guanine nucleotide-exchange factor; HSP90, heat shock protein 90; MYC2, transcription factor MYC2.

Most DEGs associated with plant-pathogen interaction were downregulated after AL12 inoculation (Figure 5). Among the genes related to pathogen-associated molecular pattern (PAMP)-triggered immunity (PTI), DEGs annotated as leucine-rich repeat (LRR) receptor-like kinase FLS2, brassinosteroid insensitive 1-associated receptor kinase 1 (BAK1), LRR receptor-like serine/threonine-protein kinase EFR, calcium-dependent protein kinase (CDPK), calmodulin/calcium-binding protein (CaM/CML), mitogen-activated protein kinase kinase kinase 1 (MEKK1), and WRKY29 tended to be downregulated. However, DEGs encoding NADPHox (Rboh) and mitogen-activated protein kinase kinase 1 (MKK1/2) tended to be upregulated in inoculated compared with control plants. Regarding effector-triggered immunity (ETI), the endophytic inoculation particularly decreased the expression of genes encoding the disease resistance proteins RPM1, RPS2, and RPS5. Additionally, defense genes such as WRKY and pathogenesis-related protein1 (PR1) also tended to be repressed after AL12 inoculation.

### *Gilmaniella* sp. AL12-Regulated Genes Involved in Signaling

**FIGURE 6:**
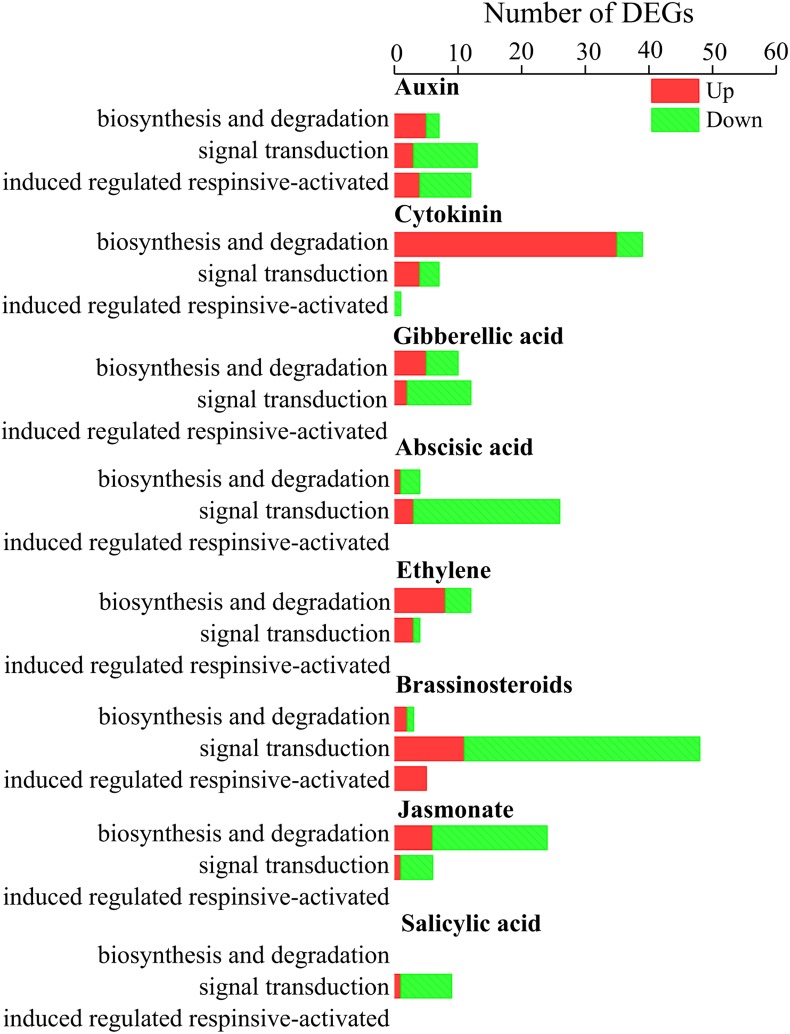
Distribution of differentially expression genes involved in hormone biosynthesis and signal transduction in shoots of *A. lancea* after the fungal endophyte *Gilmaniella* sp. AL12 inoculation. The *x* axis presents number of DEGs. The *y* axis indicates different hormone signals.

Our result shows that DEGs involved in auxin, cytokinin, and ethylene biosynthesis tended to be upregulated (Figure 6), and DEGs involved in cytokinin and ethylene signal transduction also tended to be upregulated (Figure 6). In total, 99 TF-coding genes were differentially expressed between CK and AL12 samples (Supplementary Figure S5). These DEGs were divided into 17 families, including *WRKY*, *APETALA2*/*Ethylene-Response Factors* (*AP2/ERF*), *MYB*, *basic Helix-Loop-Helix* (*bHLH*), *DELLA*, *Heat stress*, *Trilelix*, *GATA*, and *NAC*, among others. Of these families, DEGs encoding *MYB*, *Trilelix*, *and NAC* transcription factor tended to be upregulated, while many genes encoding *AP2*/*ERF*, *WRKY*, *DELLA*, *Heat stress* and *GATA* were downregulated.

### RT-qPCR Validation of Gene Expression

**FIGURE 7:**
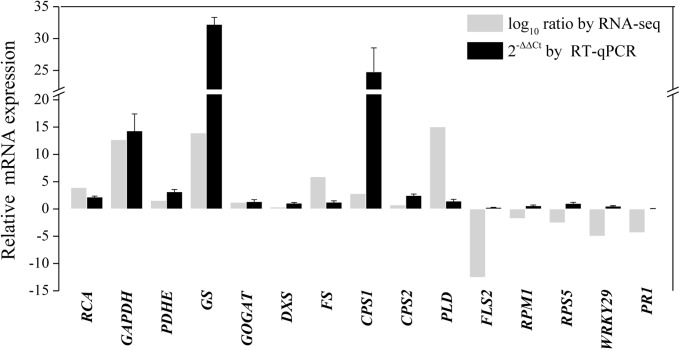
RT-qPCR validation of differentially expressed genes involved in primary metabolism, secondary metabolism, and the defense response in shoots of *A. lancea* after the fungal endophyte *Gilmaniella* sp. AL12 inoculation. Elongation factor 1 alpha gene (*EF1a*) was used as an internal reference, and the 2^−ΔΔCt^ method was used to analyze the relative mRNA expression. Values are the means of three independent experiments. Bars represent standard deviations. RCA, ribulose-1,5-bisphosphate carboxylase/oxygenase activase; GAPDH, glyceraldehyde 3-phosphate dehydrogenase; PDHE, pyruvate dehydrogenase; GS, glutamine synthetase; GOGAT, glutamate synthase; DXS, 1-deoxy-d-xylulose 5-phosphate synthase; FS, β-farnesene synthase; CPS1, β-caryophyllene synthase 1; CPS2, β-caryophyllene synthase 2; PLD, Phospholipase D; FLS2, LRR receptor-like kinase FLS2; RPM1, disease resistance protein RPM1; RPS5, disease resistance protein RPS5; WRKY29, transcription factor WRKY29; PR1, pathogenesis-related protein1.

To further validate the transcriptomic results, 15 transcripts related to primary metabolism, secondary metabolism, and defense were selected for RT-qPCR analysis (Supplementary Table S2 and Figure 7). As shown in Figure 7, the results of the RT-qPCR analysis were largely consistent with the RNA sequencing analysis, supporting the high quality of the RNA sequencing datasets. In particular, the expression of genes annotated as GAPDH, GS, and β-caryophyllene synthase CPS1 markedly increased after endophytic fungus inoculation (Figure 7).

### Shoot Proteome Changes by *Gilmaniella* sp. AL12 Inoculation Involved Diverse Biological Processes

To identify proteins that responded to the endophyte inoculation, we employed 2-DE to compare the shoot protein profiles of the CK and AL12 samples. A total of approximately 2136 protein spots were separated (Supplementary Figure S6). After optimization of the 2-DE gels, approximately 129 differentially expressed proteins with at least a two-fold change were identified (Supplementary Figure S6). Finally, 125 proteins were successfully identified via MALDI-TOF MS/MS (Supplementary Figure S7 and Supplementary Table S10). These proteins were classified into nine functional categories (Supplementary Figure S7), including “energy metabolism,” “carbohydrate metabolism,” “amino acid metabolism,” “lipid metabolism,” “defense/stress,” “plant secondary metabolism,” “signal transduction,” “transcription and translation,” and “cell growth/division.”

The expression of energy metabolism-related proteins, including ribulose bisphosphate carboxylase, ferredoxin-nitrite reductase, chloroplast oxygen-evolving enhancer protein, and phosphoenolpyruvate carboxylase, were upregulated (Table 1). Regarding carbohydrate metabolism, glyceraldehyde-3-phosphate dehydrogenase and NAD-dependent glyceraldehyde-3-phosphate dehydrogenase were up-regulated, whereas carbon catabolite repressor protein was down-regulated (Table 1). Four plant secondary metabolism-associated proteins were upregulated, including tropinone reductase, Type III polyketide synthase and cytochrome P450 CYP76F12 (Table 1).

**Table 1:** Identification of differentially expressed proteins related to energy, carbohydrate, and secondary metabolism in shoots of *A. lancea* after endophyte inoculation.

Spot	Accession no.	Protein name	MS	SC	Mr (KDa)/pI	FC
Energy metabolism						
11	AIN35032.1	AtpB (chloroplast) [*Atractylodes lancea*]	124	0.62	53.475/5.07	1.57
12	AIN35032.1	AtpB (chloroplast) [*Atractylodes lancea*]	122	0.62	53.475/5.07	1.00
13	XP_020108762.1	acetylornithine aminotransferase, chloroplastic/mitochondrial-like [Ananas comosus]	66	0.36	50.817/6.61	2.34
14	AFV93500.1	chloroplast ribulose bisphosphate carboxylase/oxygenase activase beta2, partial [Gossypium barbadense]	68	0.53	27.983/5.48	1.60
18	P28426.1	RecName: Full = Ribulose bisphosphate carboxylase large chain; Short = RuBisCO large subunit	85	0.46	51.982/6.10	2.25
19	AKG48845.1	ribulose-1,5-bisphosphate carboxylase/oxygenase large subunit, partial (chloroplast) [Desmodium triflorum]	81	0.36	49.071/6.08	4.50
20	AHW52074.1	ribulose-1,5-bisphosphate carboxylase/oxygenase large subunit, partial (chloroplast) [Cuscuta glomerata]	93	0.49	39.449/8.12	2.74
42	CAA46941.1	ferredoxin-nitrite reductase, partial [Nicotiana tabacum]	72	0.66	39.410/6.22	2.50
51	KQL23007.1	hypothetical protein SETIT_029918mg [Setaria italica](maturation RBCL)	75	0.55	49.921/7.56	2.20
59	AEO21912.1	chloroplast oxygen-evolving enhancer protein 1 [Dimocarpus longan]	77	0.51	35.386/5.85	2.08
63	XP_021983238.1	oxygen-evolving enhancer protein 1, chloroplastic [Helianthus annuus]	92	0.51	34.483/5.40	1.22
70	CAX65710.1	phosphoenolpyruvate carboxylase, partial [Stipagrostis plumosa]	61	0.57	57.090/6.62	1.31
73	CAX65709.1	phosphoenolpyruvate carboxylase, partial [Stipagrostis plumosa]	63	0.51	56.953/6.62	2.20
85	XP_017235881.1	PREDICTED: 4-hydroxy-3-methylbut-2-en-1-yl diphosphate synthase (ferredoxin), chloroplastic [Daucus carota subsp. sativus]	63	0.34	82.658/5.88	2.29
86	XP_019702089.1	PREDICTED: ATP-dependent Clp protease ATP-binding subunit CLPT1, chloroplastic-like [Elaeis guineensis]	69	0.58	26,047/9.52	1.88
87	XP_010066998.2	PREDICTED: ATP-dependent Clp protease proteolytic subunit 2, mitochondrial [Eucalyptus grandis]	64	0.48	27.713/8.48	2.16
21	AKG49084.1	ribulose-1,5-bisphosphate carboxylase/oxygenase large subunit, partial (chloroplast) [Mikania scandens]	85	0.55	48.515/5.90	0.13
22	AAV65407.1	ribulose-1,5-bisphosphate carboxylase/oxygenase large subunit, partial (chloroplast) [Baccaurea javanica]	69	0.32	52.288/6.20	0.41
25	BAJ16757.1	ribulose-1,5-bisphosphate carboxylase/oxygenase large subunit, partial (chloroplast) [Porana sp. SH-2010]	88	0.51	47.096/6.69	0.26
27	AAY16675.1	ribulose-1,5-bisphosphate carboxylase/oxygenase large subunit, partial (chloroplast) [Sclerocroton cornutus]	79	0.46	52.129/6.23	0.30
28	CAA60835.1	ribulose-1,5-bisphosphate carboxylase, partial (chloroplast) [Coopernookia strophiolata]	67	0.39	52.117/6.09	0.27
79	ASP44102.1	ribosomal protein subunit 4, partial (chloroplast) [Pohlia leucostoma]	90	0.58	22.957/10.33	0.20
92	XP_013456760.1	rubisco large subunit N-methyltransferase [Medicago truncatula]	74	0.35	39.912/9.23	0.38
94	EMT15451.1	Protochlorophyllide reductase A, chloroplastic [Aegilops tauschii]	59	0.37	36.226/9.57	0.26
Carbohydrate metabolism						
15	O04016.1	RecName: Full = Pyrroline-5-carboxylate reductase; Short = P5C reductase; Short = P5CR	68	0.42	28.986/9.04	2.74
44	AFD54987.1	beta-galactosidase [Momordica charantia]	59	0.31	81.056/8.34	1.55
52	BAJ10716.1	glyceraldehyde-3-phosphate dehydrogenase [Cladopus doianus]	72	0.41	23.904/6.12	2.69
58	EMS64835.1	putative alpha,alpha-trehalose-phosphate synthase [UDP-forming] 7 [Triticum urartu]	70	0.35	90.131/5.53	2.94
83	AIF74649.1	NAD-dependent glyceraldehyde-3-phosphate dehydrogenase short paralog, partial [Cystopteris bulbifera]	69	0.57	24.075/8.97	1.88
32	XP_020208539.1	carbon catabolite repressor protein 4 homolog 3-like [Cajanus cajan]	62	0.39	20.682/9.49	0.32
49	AAK97663.1	At1g66700/F4N21_16 [Arabidopsis thaliana] (SAM dependent carboxyl methyltransferase)	78	0.56	36.507/6.61	0.34
104	XP_013688170.1	PREDICTED: pectinesterase/pectinesterase inhibitor-like [Brassica napus]	51	0.47	10.386/6.45	0.63
125	XP_002445591.1	pectinesterase isoform X2 [Sorghum bicolor]	58	0.18	68.257/8.98	0.32
Secondary metabolism						
34	XP_016571991.1	PREDICTED: tropinone reductase-like 3 isoform X2 [Capsicum annuum]	69	0.41	23.370/8.81	4.99
56	BAF26158.2	Os10g0177300 [Oryza sativa Japonica Group] (Type III polyketide synthase family protein)	77	0.42	44.280/6.08	2.09
84	XP_013752435.1	PREDICTED: caffeic acid 3-O-methyltransferase-like [Brassica napus]	63	0.35	43.001/5.30	2.70
103	AOE22893.1	cytochrome P450 CYP76F12 [Vitis vinifera]	62	0.24	57.215/6.55	2.60

*The spot number corresponds to the number shown in Supplementary Figure S6. MS, mascot score; SC, sequences coverage; MW, molecular weight; pI, isoelectric points; FC, fold change.*

Among the 30 proteins involved in signal transduction, 17 were upregulated. These proteins included membrane-associated protein, protein kinase, protein phosphatase, abscisic acid (ABA) responsive protein, gibberellin (GA) biosynthesis related proteins, indoleacetic acid (IAA) biosynthesis related proteins, and a few transcription factors (Supplementary Table S10). The other 13 downregulated proteins incorporated pentapeptide, ethylene biosynthesis-related protein, and ubiquitin conjugate factors, among others (Supplementary Table S10). Additionally, AL12-regulated proteins involved in defense/stress response, amino acid and lipid metabolism, transcription and translation, cell growth, and other function were differentially regulated (Supplementary Table S10).

## Discussion

**FIGURE 8:**
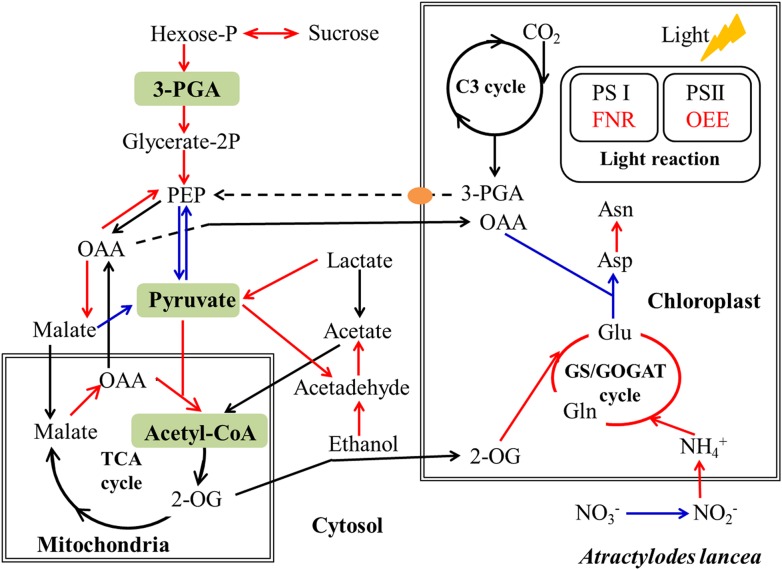
Simplified schematic summary of metabolic changes in shoots of *A. lancea* after the fungal endophyte *Gilmaniella* sp. AL12 inoculation. DEGs with significant differences in endophyte inoculated plants are shown as colored arrows (upregulated DEGs in red and downregulated DEGs in blue). The yellow ellipse indicates the phosphate translocator (TPT). Letters in green blocks represent precursor substances of terpenoid biosynthesis. C3 cycle, Benson-Calvin cycle; GS/GOGAT, glutamine synthetase (GS)/glutamate synthase (GOGAT); TCA cycle, tricarboxylic acid cycle; PS I, photosystem I; PS II, photosystem II; 3-PGA, glycerate-3-phosphate; OAA, oxaloacetate; 2-OG, 2-oxoglutarate; FNR, ferredoxin-NADP reductase; OEE, photosystem II oxygen-evolving enhancer protein.

The pharmaceutical value of medicinal plants relies on the accumulation of active pharmaceutical ingredient, and guaranteeing the yield and quality of these herbs is the main challenge (Yang et al., 2012). Endophytes have been proven to exert multiple effects on their host plants, including plant growth promotion, secondary metabolite biosynthesis promotion, stress resistance enhancement (Ludwig-Müller, 2015; Yang et al., 2016; Dastogeer et al., 2018). The advantages provided by the endophyte fungus AL12 on the host plant *A. lancea* were an improved plant biomass and increased sesquiterpenoids content (Yuan et al., 2016b). In this study, transcriptomics and proteomics were employed to analyze how endophytic fungi affect the regulation of transcription and translation in *A. lancea*. Compared to the reported endophyte associations (Doehlemann et al., 2008; Kawahara et al., 2012; Morán-Diez et al., 2012; Perazzolli et al., 2012; De Cremer et al., 2013; Zouari et al., 2014; Adolfsson et al., 2017; Chen W. et al., 2017; Dinkins et al., 2017; Hao et al., 2017; Bajaj et al., 2018), the endophyte *Gilmaniella* sp. AL12 resulted in 2.7% differential gene expression, thus demonstrating a greater impact on their host than most other endophytes (Supplementary Table S11). During the plant life cycle, a dynamic trade-off between growth and defense is necessary for plant resource assignment in response to multiple developmental cues and environmental stimuli (Hou et al., 2013). In contrast to the common downregulated plant-pathogen interactions and most phytohormone signaling events, *Gilmaniella* sp. AL12 upregulated genes involved in primary and secondary metabolism (Figure 2–4, 8). Consistent with the improved cytokinin content of *A. lancea* after AL12 inoculation (Yuan et al., 2016b), DEGs involved in cytokinin biosynthesis and signal transduction both tended to be upregulated (Figure 6), which may induce cell division, accelerate chlorophyll biosynthesis, and delay leaf senescence (Cortleven and Schmülling, 2015). In addition, ethylene induced by the endophyte mediates sesquiterpenoid biosynthesis in *A. lancea* (Yuan et al., 2016a). Based on these results, we propose that colonization by the endophytic fungus *Gilmaniella* sp. AL12 will shift plant metabolism from defense to growth, allowing the host plant to utilize limited energy for carbon assimilation and, thus, to achieve an increased biomass and sesquiterpenoid content in *A. lancea*.

It is acknowledged that plant primary metabolism and secondary metabolism required large amounts of energy. One way to support the increased energy demands is to enhance the carbon assimilation efficiency (Rozpądek et al., 2015, 2019). We have previously observed that the net photosynthesis rate and contents of chlorophyll, rubisco, and soluble carbohydrate in *A. lancea* increase after AL12 inoculation (Yuan et al., 2016b). In the present study, phenomenological fluxes per cross section of the host plant was improved after AL12 inoculation (Figure 1D), indicating the improvement of PS II reaction center performance in *A. lancea*. As shown by the transcriptome and proteome results, endophyte inoculation would enhance NADPH production and CO_2_ assimilation (Table 1, Figure 3, 8 and Supplementary Figure S8), thus explaining the positive effects of endophyte inoculation on plant photosynthesis. Similarly, *Dactylis glomerate* inoculated with *Epichloë typhina*, *Medicago truncatula*, and *Populus alba* inoculated with AMF all displayed a similar photosynthesis performance with improved plant light-driven energy production efficiency, Rubisco content, CO_2_ assimilation and PS II photochemistry efficiency (Lingua et al., 2008; Aloui et al., 2009; Cicatelli et al., 2010, 2012; Rozpądek et al., 2015, 2019). Although *Gilmaniella* sp. AL12, *Epichloë typhina* and AMF belong to distinct fungal species, they all provide a benefit, such as improving similar photosynthesis apparatuses of their host plant. Plant photosynthesis and respiration processes exist with each other interdependently and share adenosine diphosphate (ADP) and NADP^+^ or NAD^+^. Additionally, O_2_ produced by photosynthesis can be utilized by the respiration process, and CO_2_ produced by respiration can also be assimilated by photosynthesis. As speculated based on the results of this study (Table 1 and Figure 3), NADPH and O_2_ production by chloroplasts, as well as NADH dehydrogenation and triphosadenine (ATP) production by mitochondria, were improved after AL12 inoculation, supporting energy storage for plant metabolism. The TCA cycle mainly converts pyruvate to malate to produce ATP, incorporating citric acid biosynthesis, oxidation and decarboxylation, and OAA regeneration (Fernie et al., 2004). *Epichloë typhina* has been shown to markably strengthen NADPH-MDH enzyme activity in its host *Dactylis glomerate* (Rozpądek et al., 2015). Likewise, the endophytic fungus *Gilmaniella* sp. AL12 upregulated genes encoding MDH and pyruvate dehydrogenase (PDHE) (Figure 3), thus contributing to acetyl-CoA biosynthesis and OAA regeneration (Figure 8). Acetyl-CoA and pyruvate biosynthesis were improved in *A. lancea* after endophyte inoculation (Figure 8), indicating that more precursor substances and energy were available for plant secondary metabolite biosynthesis (Nema et al., 2013). As the precursor substances of terpenoid biosynthesis (Chen F. et al., 2017), pyruvate and acetyl-CoA content increased after AL12 inoculation (Yuan et al., 2016b). Additionally, in contrast to the general downregulation of fatty acid biosynthesis (Supplementary Table S4), genes associated with terpenoid biosynthesis were upregulated after AL12 inoculation (Supplementary Figure S3), indicating that a greater amount of acetyl-CoA would be diverted into terpene biosynthesis.

Plant terpenoids are derived from isopentenyl diphosphate (IPP) and dimethylallyl diphosphate (DMAPP) synthesized through the mevalonic acid (MVA) and the 2-C-methyl-D-erythritol-4-phosphate (MEP) pathway (Vattekkatte et al., 2018). The different molecular rates of isopentenyl diphosphate and dimethylallyl diphosphate condensation lead to geranyl diphosphate (GPP) for monoterpene, farnesyl diphosphate (FPP) for sesquiterpene and geranylgeranyl diphosphate (GGPP) for triterpene biosynthesis (Dudareva et al., 2013; Vattekkatte et al., 2018). Consistent with the increased sesquiterpenoids content in *A. lance*a shoots (Figure 1A), DEGs associated with terpenoid backbone biosynthesis and sesquiterpene biosynthesis were increased (Supplementary Figure S3). Unfortunately, we did not detect any spot identified associated with terpene backbone biosynthesis or sesquiterpenoids biosynthesis in proteome (Table 1). Considering that there is no database of *A. lancea* or related genus in NCBI, we identified proteins using NCBI green plant database. Thus, we considered that maybe some proteins related to secondary metabolism may not identified exactly. Yet despite that, comparative transcriptomics showed that genes related to terpene backbone biosynthesis and sesquiterpenoids biosynthesis tended to be upregulated in shoots of *A. lancea* after the endophyte inoculation (Supplementary Figure S3). In this study, high-quality reads were assembled *de novo* by using the Trinity program. The results showed that genes annotated as β-farnesene synthase and β-caryophyllene synthase were upregulated after AL12 inoculation, as shown by the transcriptome (Supplementary Figure S4) and RT-qPCR results (Figure 7). It is known that one terpene synthase can catalyze formation more than one terpene (Dudareva et al., 2013). For example, TPS21 and TPS11 from *Arabidopsis thaliana* are responsible for the biosynthesis of more than 20 sesquiterpenoids in flower fragrance (Tholl et al., 2005). Additionally, OkBCS of *Ocimum* can catalyze the production of β-caryophyllene and α-humulene (Jayaramaiah et al., 2016). The main sesquiterpenoids of *A. lancea* incorporate β-caryophyllene, zingiberene, β-sesquiphellandrene, caryophyllene oxide, hinesol, β-eudesmol and atractylone (Yuan et al., 2016b). Considering the multifunction of sesquiterpene synthase, we propose that β-farnesene synthase and β-caryophyllene synthase are crucial terpene synthases induced by AL12 to supply higher sesquiterpenoid contents in *A. lancea*. Further experiments will include the functional characterization of these sesquiterpene synthases, and how the fungal endophyte regulate genes coding for these sesquiterpene synthases.

The accumulation of secondary metabolites is regulated by cross-talking signaling cascades. Our previous studies have demonstrated that the endophyte AL12 activates multiple signals to induce plant sesquiterpenoids accumulation (Wang et al., 2011; Ren and Dai, 2012, 2013; Yuan et al., 2016a). In addition, mannan-binding lectin has been proposed to recognize mannan of *Gilmaniella* sp. AL12, thus causing signal transduction (Chen et al., 2016). It is worth noting that 32 DEGs encoding PLD tended to be upregulated after AL12 inoculation in the present study (Supplementary Table S8). PLD can hydrolyse phospholipids into phospholipid acid, which acts as an important molecular signal mediating heat stress-induced triterpene ganoderic acid biosynthesis in *Ganoderma lucidum* (Liu et al., 2017). These results indicate that phospholipid acid may be an essential signaling molecule in *A. lancea* after AL12 inoculation. Additionally, metabolomics and transcriptomics are required to study whether PLD or phospholipid signaling in *A. lancea* are activated and whether they mediate sesquiterpenoid biosynthesis after AL12 inoculation, which will help to understand their roles in inducing the biosynthesis of the secondary metabolites.

In contrast to the enhanced primary and secondary metabolism most genes involved in the plant-pathogen interaction were downregulated (Figure 2, 5). Plants evolve pattern recognition receptors (PRRs) to recognize evolutionarily conservative PAMPs, triggering the host MAPK cascade (Figure 5). Then, MAPK activates plant hormone signal transduction to integrate diverse aspects of PTI (Cui et al., 2015; Pandey et al., 2016). These optional signaling events will give rise to various immune responses (Akira and Hemmi, 2003). Furthermore, effectors produced by the fungus are also known to inhibit MAPK cascade signaling or block plant-resistant protein expression, thus suppressing plant basic immune response PTI (Zhang et al., 2012; Meng and Zhang, 2013). Fungi deploy these co-evolved effectors to modify host plant cell processes or to establish biotrophic or other types of symbiotic relationship with the host (Le Fevre et al., 2015). In turn, fungal effectors can be recognized by plant disease resistance proteins (R proteins) and thus trigger plant ETI, which usually causes the plant hypersensitive response (HR) (Jones and Dangl, 2006; Liu et al., 2007). With endophytic fungal AL12 inoculation, genes annotated as plant PRRs, including LRR receptor-like kinase FLS2, BAK1 and EFR, tended to be downregulated (Figure 5), indicating a weakened PAMP recognition ability of the host plant. Furthermore, genes involved in the response of PAMPs or effectors were also repressed (Figure 5), suggesting that endophyte inoculation suppressed host immune reactions. As shown in our previous study (Yang and Dai, 2013), AL12 could successfully colonize the leaves of *A. lancea*, resulting in an altered hyphal morphology such as smooth and unbranched hyphae. In addition, slightly pointed hyphal tips of the endophyte also appears similar as the ends of a nematode (Yang and Dai, 2013). Considering that genes related to PTI and ETI of *A. lancea* were downregulated after AL12 inoculation (Figure 5) and that the hyphae of the endophyte were altered morphologically (Yang and Dai, 2013), we deduced that either masked fungal PAMPs were not detected by the host plant, or that the plant defense response was suppressed by the host or the endophyte (Dupont et al., 2015), potentially contributing to the compatible association of *Gilmaniella* sp. AL12 with *A. lancea*.

Given the apparent downregulation of genes involved in the plant-pathogen interaction, endophyte inoculation would enhance the pathogen susceptibility of the host plant. However, this is not the actual fact. Although plant defense-related genes of *Perennial ryegrass* were down-regulated after *Epichloë festucae* var. *lolii* infection, its resistance to fungal pathogens was enhanced due to the production of phenolic compounds (Tian et al., 2008; Pańka et al., 2013; Dupont et al., 2015). Similarly, *Gilmaniella* sp. AL12 upregulated genes involved in the biosynthesis of phenylpropanoids and terpenes (Figure 4). Terpenoids are frequently used as phytoalexins, the accumulation of which are enhanced by biotic or abiotic stress (Schmelz et al., 2014; Vaughan et al., 2015). The sesquiterpenoid phytoalexins such as gossypol, hemigossypolone and heliocides can provide defense mechanisms against pathogens and herbivores of cotton (Yang C. Q. et al., 2013). Additionally, β-caryophyllene can defend against *Helicoverpa armigera* and *Pseudomonas syringae* pv. t*omato* DC3000 (Huang et al., 2012; Singh et al., 2014). Similarly, the monoterpene S-limonene and triterpene momilactones or oryzalexins function as defensive metabolites against *Magnaporthe oryzae* or *Magnaporthe grisea* (Toyomasu et al., 2008; Chen et al., 2018). Therefore, although defense-related genes of *A. lancea* were downregulated after *Gilmaniella* sp. AL12 inoculation, the enhanced synthesis of phenylpropanoids and terpenoids may function as defensive strategy against other microbial pathogens or herbivores, functioning as a possible competitive exclusion tactic of the endophyte.

**FIGURE 9:**
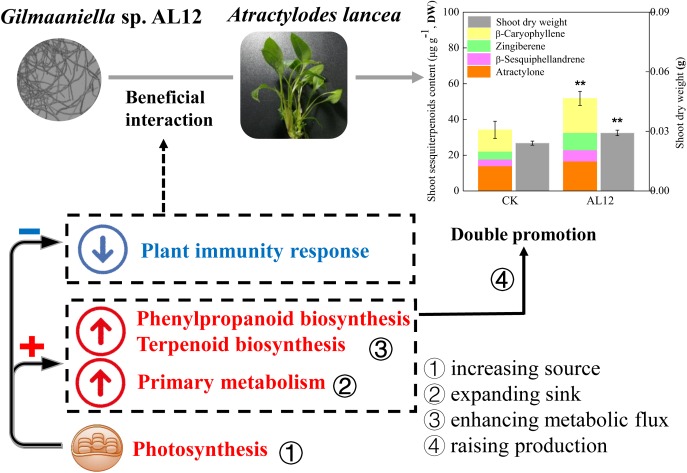
Simplified schematic summary of the “double-promotion” effect on *A. lancea* bring by the fungal endophyte *Gilmaniella* sp. AL12. The fungal endophyte *Gilmaniella* sp. AL12 weakened the plant immune response of *A. lancea*, thus may contributing to plant-endophyte beneficial interaction. Additionally, endophyte-plant association increased production (biomass and sesquiterpenoid content) by increasing the source (photosynthesis), expanding the sink (glycolysis and the TCA cycle), and enhancing metabolic flux (sesquiterpenoids biosynthesis-related genes) in *A. lancea*.

In this study, shoots of *A. lancea* were sterile, and were appropriate for investigating the effect of the fungal endophyte on plantlets without the distraction of other biotic or abiotic factors, thus helping to understand plant-endophyte interactions. The above results showed that the fungal endophyte *Gilmaniella* sp. AL12 weakened the plant immune response, which might contribute to its successful and stable colonization of the host plant (Figure 9). The decreased plant immunity resulted in large amounts of energy for plant primary or secondary metabolism. Moreover, the fungal endophyte improved photosynthesis, carbon fixation, carbohydrate transformation, and the conversion from pyruvate to acetyl-CoA in the host plant, indicating that the endophyte promoted the accumulation of precursors for terpenoid biosynthesis. In addition, the fungal endophyte specifically induced sesquiterpenoid biosynthesis by regulating genes involved in sesquiterpenoid biosynthesis and related signaling events in *A. lancea*. Thus, we propose that the fungal endophyte-plant association increased production (biomass and sesquiterpenoid content) by increasing the source (photosynthesis), expanding the sink (glycolysis and the TCA cycle), and enhancing metabolic flux (sesquiterpenoids biosynthesis-related genes) in *A. lancea* (Figure 9). Consistent with the “double promotion” in this study, the endophytic fungi AL12 promotes plant growth and sesquiterpenoid accumulation within two years of growth in field experiments. Further studies will be conducted to investigate whether pre-inoculation with the fungal endophyte *Gilmaniella* sp. AL12 regulate metabolism and immunity of *A. lancea* in the field experiments, which will help to understand plant-endophyte interactions, and will contribute to the application of the fungal endophyte in cultivating of *A. lancea*.

Medicinal plants are rich in active compounds such as artemisinin (Sharma and Agrawal, 2013) and ginseng saponin (Wei et al., 2018), which are important sources of modern drugs. You-You Tu, the mother of artemisinin, was awarded Nobel Prize in medicine in 2015. The development of the medicinal plants gradually becomes a focused issue, and medicinal plants-endophytes interactions have received much attentions. A review of the medicinal plant microbiome has shown that the secondary metabolite content of medicinal herbs depends on where they are cultivated, which can be partly ascribed to different rhizospheric or endophytic microbes associated with their cultivation location (Köberl et al., 2013; Huang et al., 2018). Plantlets of *A. lancea* grown in the Maoshan area of southeast China represent the geo–authentic medicinal plant, with a much greater sesquiterpenoid content and diversity than *A. lancea* in other cultivation area (Zhou et al., 2018). The endophytic fungus *Gilmaniella* sp. AL12 was isolated from the stem of the geo–authentic *A. lancea*, and the compatible association contributes some benefits to the host plant, including the promotion of plant growth and sesquiterpenoid content, and the prevention of root rot (Wang et al., 2011; Ren and Dai, 2012, 2013; Wang et al., 2012; Yuan et al., 2016a,b; Ren et al., 2017). Whether *Gilmaniella* sp. AL12 is a key member of the core microbiome of geo–authentic *A. lancea* remain unanswered. In this regard, our previous studies have shown that prioritizing endophytic *Gilmaniella* sp. AL12 inoculation enhances the diversity and size of phyllospheric microbial communities of *A. lancea* by increasing the soluble sugar content in the rhizosphere (Yang T. et al., 2013). Given that the endophyte provided physiological and biochemical benefits to the host and affected host phyllospheric microbial communities, we more broadly propose a beneficial association of endophytic *Gilmaniella* sp. AL12 with *A. lancea* as a potential model for endophytic fungi-medicinal herb interaction. The endophyte-*A. lancea* association contributes to medicinal herb cultivation and helps to further clarify plant-endophyte interactions.

## Conclusion

In summary, this study showed that the fungal endophyte *Gilmaniella* sp. AL12 weakened the plant immune response of *A. lancea*, thus may contributing to the beneficial plant-endophyte interaction. Additionally, endophyte-plant association increased production (biomass and sesquiterpenoid content) by increasing the source (photosynthesis), expanding the sink (glycolysis and the TCA cycle), and enhancing metabolic flux (sesquiterpenoids biosynthesis-related genes) in *A. lancea*. This study revealed the regulation of *Gilmaniella* sp. AL12 on plant metabolism and related regulatory processes in shoots of *A. lancea* at the transcriptional and translational level. This study provides a theoretical basis for medicinal herb cultivation and helps to further clarify plant-endophyte interactions.

## Data Availability

The raw data supporting the conclusions of this manuscript will be made available by the authors, without undue reservation, to any qualified researcher.

## Author Contributions

C-CD and JY designed the experiments, analyzed the data, and wrote the manuscript. P-XC and M-JT helped extract plant RNA and protein. WZ, KS, and XL helped supervise the manuscript writing. All the authors read and approved the final manuscript.

## Conflict of Interest Statement

The authors declare that the research was conducted in the absence of any commercial or financial relationships that could be construed as a potential conflict of interest.

## Footnotes

1 http://pathways.embl.de/iPath2.cgi#

## Abbreviations

*A. lancea*: *Atractylodes lancea*
AL12: *Gilmaniella* sp. AL12
AMF: arbuscular mycorrhizal fungi
ATP: triphosadenine
BAK1: brassinosteroid insensitive 1-associated receptor kinase 1
DEGs: differentially expressed genes
DPI: days post-AL12 inoculation
EFR: LRR receptor-like serine/threonine-protein kinase EFR
ETI: effector triggered immunity
FPKM: the fragments per kilobase of transcript per million mapped reads
GC: gas chromatography
GO: gene ontology database
KEGG: Kyoto Encyclopedia of Genes and Genomes database
LRR: leucine-rich repeat
MDH: malate dehydrogenase
NCBI: National Center for Biotechnology Information
OAA: oxaloacetate
PAMPs: pathogen-associated molecular patterns
Pi: orthophosphate
PLD: phospholipase D
PS II: photosystem II
PTI: PAMP triggered immunity
RT-qPCR: real-time quantitative PCR
TCA cycle: tricarboxylic acid cycle
2-DE: two-dimensional gel electrophoresis.
