# Supplementary material for: Comparative Transcriptomics and Proteomics of Atractylodes lancea in Response to Endophytic Fungus Gilmaniella sp. AL12 Reveals Regulation in Plant Metabolism
Source: Front Microbiol. 2019 May 28;10:1208. doi: 10.3389/fmicb.2019.01208 (PMC6546907; doi:10.3389/fmicb.2019.01208)
Supplement: Supplementary file 1 [file Data_Sheet_1.docx]

Supplementary Material

# Supplementary Table S1. Fluorescence parameter and calculation formula.

| Fluorescence parameter calculation | Fluorescence parameter |
| --- | --- |
| F_o_ | the minimal fluorescence yield |
| F_m_ | the maximum fluorescence yield |
| F_v_ = F_t_ -F_o_ | the relative variable fluorescence yield |
| F_j_ = F_2ms_ - F_o_ | the relative variable fluorescence yield at j point (t = 2 ms) |
| F_v_/F_m_ = φP_o_ = 1-( F_o_ / F_m_ ) | the maximum quantum yield of primary photochemistry |
| φE_o_ = φP_o_ × [1-(F_j_-F_o_)/(F_m_- F_o_)] | quantum yield for electron transport (t = 0) |
| t_Fm_ | the time required from dark adaptation to maximum fluorescence |
| ABS/CS_m_ = F_m_ | absorption flux of photons per cross section (t = t_Fm_) |
| TR_o_/CS_m_ = φP_o_ × (ABS/CS_m_) | phenomenological fluxes for trapping per cross section (t = t_Fm_) |
| ET_o_/CS_m_ = φE_o_ × (ABS/CS_m_) | potential electron transport per cross section (t = t_Fm_) |
| DI_o_/CS_m_ = (ABS/CS_m_) - (TR_o_/CS_m_) | dissipation per cross section (t = t_Fm_) |

# Supplementary Table S2. Primers of 14 selected genes related to primary metabolism, secondary metabolism and the defence response in *A. lancea*.

| **Gene** | **Gene ID** | **Log_10_**  **(AL12/CK)** | **Forward** | **Reverse** |
| --- | --- | --- | --- | --- |
| ***RCA*** | Unigene10185_All | 3.94 | TGCTGCTCCTCGACGACCTC | GGAAGCGACAGAGAAGCATAGGC |
| ***GAPDH*** | Unigene5281_All | 12.69 | GACGACGGCTTGGAGGTCAATG | TCGGCGGAAGGAGCAGAGATG |
| ***PDHE*** | CL16265.Contig2_All | 1.56 | CAGTAGGTGGTTCTCGAACGGTTG | TGACGCTCCGACCTCTGATGTG |
| ***GS*** | Unigene37306_All | 13.94 | CCACAAGGTCCACGAGCCATG | CCAGCACCAACACCGCAGTAG |
| ***GOGAT*** | Unigene41542_All | 1.21 | CCATCACCACCAAGCGAGAC | TGTGCACCCTCAACAAGCAA |
| ***DXS*** | CL16217.Contig18_All | 0.38 | CAACTCTCCATCTCCATGCCTCAC | ATCGCCATCTTCCGTTCTTCAGC |
| ***FS*** | CL1221.Contig3_All | 5.91 | TTGGCATCAAGGCCAAGGAT | GTAACGCACTGCCTCTAGCC |
| ***CPS1*** | CL1760.Contig2_All | 2.83 | ACCTGCAAACTGCACAGGAA | ACCAACAACAGAGGAGCACA |
| ***CPS2*** | CL1760.Contig3_All | 0.75 | GTTGGAGTTGGACGAGTTGTGGAG | ATGCTATGCCGAGACGTTGGATTG |
| ***PLD*** | CL1524.Contig1_All | 15.07 | CAGTGTAGGTGACGCCGTTGAAG | TCTGCCTCTGCCTGGTCCAAC |
| ***FLS2*** | Unigene26790_All | -12.56 | AGGAAAGATGCGGGCGAACT | TGGCTACTCAGGTGGATGGC |
| ***RPM1*** | Unigene28171_All | -4.99 | ATCGCTATGTGGTCAAGGTGTGC | CATCAATGCTGGCCTGGAGACTG |
| ***RPS5*** | Unigene24052_All | -4.34 | TTCCAGGACTTCCACAGCGTATTG | GGTGTCATCCTTGAGCAGCCTATG |
| ***WRKY29*** | CL1374.Contig13_All | -1.77 | AGGATGTCCGGTGAGGAAGCG | GGCGTTGGAGCGTGATGGTG |
| ***PR1*** | Unigene67747_All | -2.55 | AGGTGTGGATGCGGTGAACTTG | TCACGAACCACGCACCACTATTG |
| ***EF1α*** |  |  | CAGGCTGATTGTGCTGTTCTTA | TGTGGCATCCATCTTGT |

Abbreviations: ribulose-1,5-bisphosphate carboxylase/oxygenase activase (RCA), glyceraldehyde 3-phosphate dehydrogenase (GAPDH), pyruvate dehydrogenase (PDHE), glutamine synthetase (GS), glutamate synthase (GOGAT), 1-deoxy-d-xylulose 5-phosphate synthase (DXS), β-farnesene synthase (FS), β-caryophyllene synthase (CPS1 and CPS2), phospholipase D (PLD), LRR receptor-like kinase FLS2 (FLS2), disease resistance protein RPM1 (RPM1), disease resistance protein RPS5 (RPS5), transcription factor WRKY29 (WRKY29), and pathogenesis-related protein1 (PR1). Elongation factor 1 alpha gene (*EF1a*) was used as an internal reference.

# Supplementary Table S3. Distribution of DEGs involved in transcription, translation, replication and repair, and folding sorting and degradation.

| **Pathway** | **Up DEGs** | **Down DEGs** |
| --- | --- | --- |
| RNA polymerase | 12 | 24 |
| Basal transcription factors | 3 | 1 |
| Spliceosome | 57 | 38 |
| Aminoacyl-tRNA biosynthesis | 1 | 0 |
| Ribosome biogenesis in eukaryotes | 56 | 6 |
| Ribosome | 4 | 23 |
| RNA transport | 65 | 28 |
| mRNA surveillance pathway | 18 | 22 |
| DNA replication | 5 | 5 |
| Base excision repair | 3 | 4 |
| Nucleotide excision repair | 6 | 7 |
| Mismatch repair | 3 | 4 |
| Homologous recombination | 34 | 4 |
| Non-homologous end-joining | 6 | 0 |
| RNA degradation | 49 | 12 |
| Proteasome | 4 | 0 |
| Protein export | 4 | 4 |
| Ubiquitin mediated proteolysis | 9 | 24 |
| SNARE interactions in vesicular transport | 1 | 7 |
| Protein processing in endoplasmic reticulum | 39 | 30 |

# Supplementary Table S4. Distribution of DEGs involved in nucleotide metabolism, glycan biosynthesis and metabolism, and lipid metabolism.

| **Pathway** | **Up DEGs** | **Down DEGS** |
| --- | --- | --- |
| Purine metabolism | 17 | 31 |
| Pyrimidine metabolism | 19 | 30 |
| N-Glycan biosynthesis | 1 | 8 |
| Other glycan degradation | 6 | 9 |
| Other types of O-glycan biosynthesis | 0 | 1 |
| Glycosaminoglycan degradation | 3 | 1 |
| Glycosylphosphatidylinositol(GPI)-anchor biosynthesis | 3 | 11 |
| Glycosphingolipid biosynthesis – ganglio series | 3 | 0 |
| Fatty acid biosynthesis | 3 | 4 |
| Fatty acid elongation | 0 | 11 |
| Fatty acid metabolism | 21 | 0 |
| Cutin suberine and wax biosynthesis | 15 | 18 |
| Steroid biosynthesis | 6 | 0 |
| Glycerolipid metabolism | 4 | 7 |
| Glycerophospholipid metabolism | 42 | 33 |
| Ether lipid metabolism | 39 | 20 |
| Arachidonic acid metabolism | 0 | 3 |
| Linoleic acid metabolism | 1 | 22 |
| alpha-Linolenic acid metabolism | 6 | 20 |
| Sphingolipid metabolism | 3 | 0 |
| Biosynthesis of unsaturated fatty acids | 2 | 15 |

# Supplementary Table S5. Distribution of DEGs involved in energy metabolism and carbohydrate metabolism.

| **Pathway** | **Up DEGs** | **Down DEGS** |
| --- | --- | --- |
| Oxidative phosphorylation | 9 | 5 |
| Photosynthesis | 2 | 0 |
| Photosynthesis - antenna proteins | 0 | 3 |
| Carbon fixation in photosynthetic organisms | 3 | 6 |
| Nitrogen metabolism | 10 | 2 |
| Sulfur metabolism | 1 | 2 |
| Glycolysis / Gluconeogenesis | 31 | 4 |
| Citrate cycle (TCA cycle) | 4 | 0 |
| Pentose phosphate pathway | 1 | 4 |
| Pentose and glucuronate interconversions | 23 | 13 |
| Fructose and mannose metabolism | 5 | 8 |
| Galactose metabolism | 15 | 3 |
| Ascorbate and aldarate metabolism | 21 | 4 |
| Starch and sucrose metabolism | 35 | 30 |
| Amino sugar and nucleotide sugar metabolism | 11 | 8 |
| Inositol phosphate metabolism | 1 | 1 |
| Pyruvate metabolism | 9 | 6 |
| Glyoxylate and dicarboxylate metabolism | 2 | 1 |
| Propanoate metabolism | 4 | 2 |
| Butanoate metabolism | 1 | 6 |

# Supplementary Table S6. Distribution of DEGs involved in amino acid metabolism.

| **Pathway** | **Up DEGs** | **Down DEGS** |
| --- | --- | --- |
| Alanine aspartate and glutamate metabolism | 8 | 3 |
| Glycine serine and threonine metabolism | 5 | 1 |
| Cysteine and methionine metabolism | 18 | 8 |
| Valine leucine and isoleucine degradation | 4 | 2 |
| Valine leucine and isoleucine biosynthesis | 1 | 2 |
| Lysine biosynthesis | 2 | 1 |
| Lysine degradation | 4 | 0 |
| Arginine and proline metabolism | 14 | 9 |
| Histidine metabolism | 3 | 2 |
| Tyrosine metabolism | 33 | 4 |
| Phenylalanine metabolism | 36 | 9 |
| Tryptophan metabolism | 5 | 3 |
| Phenylalanine tyrosine and tryptophan biosynthesis | 8 | 3 |
| beta-Alanine metabolism | 5 | 0 |
| Taurine and hypotaurine metabolism | 1 | 1 |
| Cyanoamino acid metabolism | 6 | 6 |
| Glutathione metabolism | 2 | 10 |

# Supplementary Table S7. Distribution of DEGs involved in the metabolism of cofactors and vitamins.

| **Pathway** | **Up DEGs** | **Down DEGS** |
| --- | --- | --- |
| Ubiquinone and other terpenoid-quinone biosynthesis | 11 | 2 |
| Riboflavin metabolism | 2 | 0 |
| Vitamin B6 metabolism | 3 | 0 |
| Nicotinate and nicotinamide metabolism | 1 | 0 |
| Pantothenate and CoA biosynthesis | 3 | 2 |
| Porphyrin and chlorophyll metabolism | 2 | 8 |
| Lipoic acid metabolism | 0 | 1 |
| Folate biosynthesis | 0 | 1 |

# Supplementary Table S8. DEGs annotated as phospholipase D (KO_id: K01115).

| **GeneID** | **Log_10_ (AL12 vs CK)** |
| --- | --- |
| CL16358.Contig2_All | 18.58 |
| CL16358.Contig5_All | 16.56 |
| Unigene58779_All | 16.01 |
| CL1524.Contig1_All | 15.07 |
| Unigene3206_All | 14.27 |
| CL1524.Contig2_All | 13.61 |
| Unigene3032_All | 12.72 |
| CL16358.Contig4_All | 12.68 |
| CL3674.Contig2_All | 6.33 |
| CL14007.Contig2_All | 6.08 |
| CL5282.Contig1_All | 5.43 |
| Unigene46382_All | 4.49 |
| CL5282.Contig4_All | 4.21 |
| CL5282.Contig6_All | 4.12 |
| Unigene62508_All | 4.09 |
| CL5282.Contig5_All | 4.09 |
| Unigene51352_All | 4.07 |
| Unigene58288_All | 3.70 |
| Unigene32885_All | 3.66 |
| CL5282.Contig3_All | 3.59 |
| CL5282.Contig2_All | 3.44 |
| CL4160.Contig1_All | 2.75 |
| CL3879.Contig1_All | 2.34 |
| CL3146.Contig2_All | 2.26 |
| Unigene30104_All | 2.03 |
| Unigene34938_All | 1.72 |
| CL2721.Contig13_All | 1.67 |
| Unigene50742_All | 1.32 |
| Unigene34532_All | 1.13 |
| CL5488.Contig2_All | 1.08 |
| Unigene2313_All | 1.04 |
| Unigene61821_All | 1.03 |
| CL13397.Contig2_All | -1.39 |
| CL2933.Contig1_All | -1.05 |
| CL6303.Contig2_All | -12.86 |
| CL8458.Contig2_All | -1.77 |
| Unigene2599_All | -4.16 |
| Unigene32589_All | -13.00 |
| Unigene56714_All | -1.33 |
| Unigene57876_All | -1.77 |
| Unigene61113_All | -1.01 |

# Supplementary Table S9. Eighteen secondary metabolism-related KEGG pathways of AL12-regulated genes in shoots of *A. lancea*.

| **Pathway ID** | **Pathway** | **DEGs genes with pathway annotation (2014)** | **All genes with pathway annotation (57767)** | **P value** | **Q value** |
| --- | --- | --- | --- | --- | --- |
| **ko00940** | **Phenylpropanoid biosynthesis** | **81 (4.02%)** | **873 (1.51%)** | **2.61E-15** | **7.64E-14** |
| **ko00908** | **Zeatin biosynthesis** | **46 (2.28%)** | **748 (1.29%)** | **0.0001804** | **1.32E-03** |
| **ko00941** | **Flavonoid biosynthesis** | **45 (2.23%)** | **380 (0.66%)** | **1.25E-12** | **2.44E-11** |
| **ko00945** | **Stilbenoid, diarylheptanoid and gingerol biosynthesis** | **42 (2.09%)** | **300 (0.52%)** | **2.41E-14** | **5.64E-13** |
| **ko00903** | **Limonene and pinene degradation** | **24 (1.19%)** | **388 (0.67%)** | **0.0053159** | **2.49E-02** |
| **ko00944** | **Flavone and flavonol biosynthesis** | **19 (0.94%)** | **205 (0.35%)** | **0.000115** | **8.97E-04** |
| **ko00906** | **Carotenoid biosynthesis** | **18 (0.89%)** | **262 (0.45%)** | **0.0051766** | **2.49E-02** |
| **ko00904** | **Diterpenoid biosynthesis** | **14 (0.7%)** | **178 (0.31%)** | **0.0040247** | **2.05E-02** |
| ko00900 | Terpenoid backbone biosynthesis | 32 (1.59%) | 849 (1.47%) | 0.3509412 | 8.05E-01 |
| ko00950 | Isoquinoline alkaloid biosynthesis | 13 (0.65%) | 187 (0.32%) | 0.0145144 | 6.53E-02 |
| ko00960 | Tropane, piperidine and pyridine alkaloid biosynthesis | 8 (0.4%) | 177 (0.31%) | 0.2775719 | 7.66E-01 |
| ko00943 | Isoflavonoid biosynthesis | 7 (0.35%) | 76 (0.13%) | 0.0167595 | 7.26E-02 |
| ko00966 | Glucosinolate biosynthesis | 4 (0.2%) | 80 (0.14%) | 0.3047435 | 7.84E-01 |
| ko00402 | Benzoxazinoid biosynthesis | 4 (0.2%) | 85 (0.15%) | 0.3443991 | 8.05E-01 |
| ko00909 | Sesquiterpenoid and triterpenoid biosynthesis | 4 (0.2%) | 156 (0.27%) | 0.7966743 | 1.00E+00 |
| ko00905 | Brassinosteroid biosynthesis | 3 (0.15%) | 104 (0.18%) | 0.7071068 | 1.00E+00 |
| ko00902 | Monoterpenoid biosynthesis | 2 (0.1%) | 41 (0.07%) | 0.4209347 | 8.82E-01 |
| ko00942 | Anthocyanin biosynthesis | 1 (0.05%) | 8 (0.01%) | 0.247165 | 7.05E-01 |

The 8 significantly regulated pathways were screened based on Q value. The 8 pathways include Phenylpropanoid biosynthesis, Zeatin biosynthesis, Flavonoid biosynthesis, Stilbenoid, diarylheptanoid and gingerol biosynthesis, Limonene and pinene degradation, Flavone and flavonol biosynthesis, Carotenoid. biosynthesis, Diterpenoid biosynthesis.

# Supplementary Table S10. Mass spectrometric identification of differentially expressed proteins related to defence/stress, amino acid metabolism, lipid metabolism, signal transduction, transcription and translation, cell growth/division, and other unknown function in shoots of *A. lancea* after AL12 inoculation.

| **Spot** | **Accession no.** | **Protein name** | **MS** | **SC** | **Mr (Kda)^b^/pI^b^** | **Fold change** |
| --- | --- | --- | --- | --- | --- | --- |
| **Defence/Stress** | | |  |  |  |  |
| **2** | XP_022027834.1 | stromal 70 kDa heat shock-related protein, chloroplastic-like [Helianthus annuus] | 96 | 0.28 | 74.566/5.12 | 5.38 |
| **16** | AKH05125.1 | chaperonin-60 beta4 [Erodium foetidum] | 64 | 0.44 | 64.123/6.60 | 2.26 |
| **41** | XP_009784206.1 | PREDICTED: alpha-soluble NSF attachment protein-like [Nicotiana sylvestris] | 64 | 0.64 | 33.004/5.33 | 1.55 |
| **129** | XP_010051362.2 | PREDICTED: TMV resistance protein N-like [Eucalyptus grandis] | 69 | 0.38 | 141.120/5.97 | 2.57 |
| **1** | AIA22210.1 | heat shock protein 70 [Chrysanthemum indicum] | 124 | 0.63 | 71.209/5.15 | 0.39 |
| **30** | XP_021810186.1 | monodehydroascorbate reductase [Prunus avium] | 64 | 0.43 | 47.130/6.51 | 0.49 |
| **62** | XP_010245451.1 | PREDICTED: major allergen Pru ar 1-like [Nelumbo nucifera] | 69 | 0.81 | 17.853/5.35 | 3.02 |
| **80** | P_007214594.2 | cysteine-rich repeat secretory protein 4 [Prunus persica] | 71 | 0.81 | 32.771/8.27 | 0.33 |
| **Amino acid metabolism** | | |  |  |  |  |
| **67** | P_010675733.1 | PREDICTED: proline synthase co-transcribed bacterial homolog protein [Beta vulgaris subsp. vulgaris] | 67 | 0.77 | 26.872/6.09 | 2.76 |
| **50** | XP_011624183.1 | threonine dehydratase biosynthetic, chloroplastic isoform X3 [Amborella trichopoda] | 65 | 0.29 | 64.902/8.98 | 0.37 |
| **74** | EEF24738.1 | nitrilase and fragile histidine triad fusion protein, putative [Ricinus communis] | 63 | 0.65 | 33.700/6.40 | 0.44 |
| **Lipid metabolism** | | | | | | |
| **38** | XP_010258770.1 | PREDICTED: probable enoyl-CoA hydratase 2, mitochondrial [Nelumbo nucifera] | 62 | 0.29 | 35.296/8.16 | 2.19 |
| **53** | XP_020187021.1 | non-specific lipid-transfer protein 3-like [Aegilops tauschii subsp. tauschii] | 63 | 0.75 | 14.594/8.11 | 2.63 |
| **93** | XP_014518187.1 | putative lipid-transfer protein DIR1 [Vigna radiata var. radiata] | 62 | 0.96 | 11.468/9.02 | 2.12 |
| **100** | XP_017237452.1 | PREDICTED: glycolipid transfer protein 1 [Daucus carota subsp. sativus] | 64 | 0.75 | 22.776/5.65 | 2.43 |
| **54** | XP_021813071.1 | 11-beta-hydroxysteroid dehydrogenase-like 5 [Prunus avium] | 74 | 0.35 | 40.143/6.53 | 0.47 |
| **Signal transduction** | | | | | | |
| **10** | XP_009627205.1 | PREDICTED: membrane-associated protein VIPP1, chloroplastic-like [Nicotiana tomentosiformis] | 69 | 0.68 | 21.294/9.91 | 3.22 |
| **36** | XP_020208681.1 | ABA-responsive protein ABR18-like [Cajanus cajan] | 58 | 0.84 | 16.716/4.91 | 2.68 |
| **43** | XP_010473231.1 | PREDICTED: gibberellin 3-beta-dioxygenase 4-like [Camelina sativa] | 56 | 0.52 | 36.698/4.83 | 1.36 |
| **47** | XP_012698346.1 | inositol hexakisphosphate and diphosphoinositol-pentakisphosphate kinase VIP2 isoform X1 [Setaria italica] | 57 | 0.39 | 120.330/6.52 | 3.28 |
| **57** | CAE53083.1 | putative R2R3 MYB transcription factor, partial [Lolium multiflorum] | 51 | 0.4 | 6.515/9.24 | 1.19 |
| **61** | XP_008778950.2 | PREDICTED: zinc finger CCCH domain-containing protein 13-like isoform X2 [Phoenix dactylifera] | 73 | 0.35 | 94.771/5.01 | 2.44 |
| **64** | XP_017244786.1 | PREDICTED: TPR repeat-containing thioredoxin TTL1-like [Daucus carota subsp. sativus] | 66 | 0.51 | 78.089/9.42 | 1.65 |
| **65** | XP_020189970.1 | BTB/POZ and MATH domain-containing protein 2-like [Aegilops tauschii subsp. tauschii] | 60 | 0.51 | 39.937/5.38 | 1.92 |
| **66** | OVA04458.1 | Protein kinase domain [Macleaya cordata] | 71 | 0.49 | 53.790/9.68 | 1.22 |
| **71** | EOY05073.1 | RAB GTPase A5E isoform 2 [Theobroma cacao] | 58 | 0.44 | 23.545/4.94 | 1.66 |
| **76** | XP_009395211.1 | PREDICTED: probable indole-3-pyruvate monooxygenase YUCCA4 [Musa acuminata subsp. malaccensis] | 58 | 0.33 | 46.008/9.01 | 1.55 |
| **82** | XP_010103210.1 | putative protein phosphatase 2C 76 [Morus notabilis] | 63 | 0.49 | 30.018/4.59 | 1.74 |
| **88** | EOY06681.1 | Serine/threonine-protein phosphatase 4 regulatory subunit 3 isoform 2 [Theobroma cacao] | 66 | 0.29 | 100.814/5.09 | 1.67 |
| **89** | XP_008461382.1 | PREDICTED: probable receptor-like protein kinase At2g47060 [Cucumis melo] | 60 | 0.35 | 39.290/7.59 | 2.92 |
| **90** | XP_018824308.1 | PREDICTED: transcription factor MYC2-like [Juglans regia] | 57 | 0.43 | 54.491/6.16 | 1.46 |
| **97** | XP_009359782.1 | PREDICTED: probable receptor-like protein kinase At5g18500 [Pyrus x bretschneideri] | 71 | 0.43 | 55.774/8.64 | 2.46 |
| **128** | XP_010472091.1 | PREDICTED: peptidyl-prolyl cis-trans isomerase CYP19-2 [Camelina sativa] | 68 | 0.54 | 18.671/6.95 | 1.28 |
| **4** | XP_013639134.1 | PREDICTED: putative F-box/kelch-repeat protein At4g39600 [Brassica oleracea var. oleracea] | 68 | 0.89 | 16.687/9.88 | 0.30 |
| **5** | XP_018447196.1 | PREDICTED: pentatricopeptide repeat-containing protein At4g19440, chloroplastic [Raphanus sativus] | 66 | 0.32 | 91.828/8.15 | 0.39 |
| **8** | XP_012462013.1 | PREDICTED: WEB family protein At5g55860 isoform X1 [Gossypium raimondii] | 63 | 0.44 | 73.841/5.65 | 0.28 |
| **39** | XP_002280701.2 | PREDICTED: serine/threonine-protein kinase BLUS1 isoform X1 [Vitis vinifera] | 70 | 0.5 | 81.938/6.89 | 0.48 |
| **48** | XP_020597646.1 | LOW QUALITY PROTEIN: putative leucine-rich repeat-containing protein DDB_G0290503 [Phalaenopsis equestris] | 67 | 0.39 | 189.456/5.10 | 0.48 |
| **55** | XP_009759308.1 | PREDICTED: peptidyl-prolyl cis-trans isomerase CYP18-1 [Nicotiana sylvestris] | 75 | 0.76 | 17.633/7.77 | 0.30 |
| **72** | XP_013625746.1 | PREDICTED: ninja-family protein AFP4-like isoform X1 [Brassica oleracea var. oleracea] | 59 | 0.55 | 33.366/5.99 | 0.43 |
| **78** | BAQ21979.1 | 1-aminocyclopropane-1-carboxylate oxidase [Gentiana triflora] | 67 | 0.62 | 36.318/5.35 | 0.26 |
| **95** | OUS47362.1 | ras family-domain-containing protein [Ostreococcus tauri] | 64 | 0.62 | 22.432/6.40 | 0.62 |
| **105** | XP_010554643.1 | PREDICTED: coronatine-insensitive protein 1 [Tarenaya hassleriana] | 68 | 0.45 | 68.448/6.81 | 0.48 |
| **106** | XP_016497835.1 | PREDICTED: ethylene receptor 1-like, partial [Nicotiana tabacum] | 58 | 0.46 | 34.587/8.55 | 0.38 |
| **119** | OMO87719.1 | Ubiquitin conjugation factor E4, core, partial [Corchorus capsularis] | 67 | 0.34 | 58.515/5.50 | 0.43 |
| **122** | XP_010552369.1 | PREDICTED: pentatricopeptide repeat-containing protein At3g13150 [Tarenaya hassleriana] | 64 | 0.53 | 47.369/9.10 | 0.28 |
| **Transcription and translation** | | |  |  |  |  |
| **7** | XP_019425528.1 | PREDICTED: glycine-rich RNA-binding protein 6, mitochondrial [Lupinus angustifolius] | 77 | 0.88 | 15.689/9.69 | 2.04 |
| **60** | XP_020189772.1 | mediator of RNA polymerase II transcription subunit 27 [Aegilops tauschii subsp. tauschii] | 71 | 0.34 | 45.411/6.82 | 2.46 |
| **111** | XP_021671073.1 | RNA exonuclease 4-like [Hevea brasiliensis] | 64 | 0.32 | 42.496/8.60 | 1.91 |
| **35** | XP_020554150.1 | protein argonaute 2 isoform X1 [Sesamum indicum] | 74 | 0.52 | 110.916/9.25 | 0.50 |
| **46** | KYP76521.1 | Transposon Ty3-I Gag-Pol polyprotein, partial [Cajanus cajan] | 73 | 0.52 | 49.323/9.39 | 0.48 |
| **68** | XP_009775535.1 | PREDICTED: putative tRNA pseudouridine synthase Pus10 isoform X2 [Nicotiana sylvestris | 80 | 0.34 | 64.825/6.35 | 0.47 |
| **107** | XP_019236279.1 | PREDICTED: DNA repair protein XRCC2 homolog isoform X2 [Nicotiana attenuata] | 63 | 0.55 | 36.007/9.22 | 0.40 |
| **Cell growth/Division** | | |  |  |  |  |
| **102** | XP_010091188.1 | Kinesin-related protein 11 [Morus notabilis] | 66 | 0.28 | 106.583/6.06 | 1.09 |
| **9** | XP_021846436.1 | root phototropism protein 3-like [Spinacia oleracea] | 64 | 0.3 | 66.854/9.12 | 0.19 |
| **33** | XP_013458948.1 | DCD (development and cell death) domain protein [Medicago truncatula] | 73 | 0.33 | 72.811/9.72 | 0.44 |
| **Others** | | |  |  |  |  |
| **3** | BAK02933.1 | predicted protein [Hordeum vulgare subsp. vulgare] | 70 | 0.38 | 77.879/6.86 | 2.48 |
| **6** | XP_004494932.1 | PREDICTED: uncharacterized protein LOC101493817 [Cicer arietinum] （Lipase (class 3)） | 74 | 0.34 | 73.252/5.51 | 3.51 |
| **17** | KHN29105.1 | Hypothetical protein glysoja_008440 [Glycine soja] | 72 | 0.4 | 85.869/8.93 | 2.01 |
| **45** | XP_002502577.1 | predicted protein [Micromonas commoda] | 96 | 0.84 | 12.428/9.50 | 3.93 |
| **75** | XP_017604826.1 | PREDICTED: uncharacterized protein LOC108451676 [Gossypium arboreum] | 67 | 0.82 | 25.435/5.49 | 2.55 |
| **81** | XP_011467537.1 | PREDICTED: uncharacterized protein LOC101312173 isoform X2 [Fragaria vesca subsp. vesca] | 73 | 0.31 | 68.681/5.45 | 1.45 |
| **91** | XP_002503376.1 | predicted protein [Micromonas commoda] | 72 | 0.54 | 41.511/5.18 | 1.56 |
| **96** | GAV67329.1 | RVT_3 domain-containing protein [Cephalotus follicularis] | 63 | 0.52 | 24.569/9.22 | 1.48 |
| **110** | KXZ43232.1 | hypothetical protein GPECTOR_97g770 [Gonium pectorale] | 72 | 0.45 | 44.519/9.18 | 2.26 |
| **113** | XP_020182787.1 | uncharacterized protein LOC109768481 [Aegilops tauschii subsp. tauschii] | 64 | 0.24 | 182.061/6.03 | 3.33 |
| **126** | EEF30657.1 | conserved hypothetical protein [Ricinus communis] | 68 | 0.75 | 25.967/10.77 | 1.10 |
| **26** | XP_002963051.1 | hypothetical protein SELMODRAFT_61619, partial [Selaginella moellendorffii] | 73 | 0.42 | 59.555/9.63 | 0.49 |
| **29** | KCW82232.1 | hypothetical protein EUGRSUZ_C03651 [Eucalyptus grandis] | 63 | 0.53 | 25.896/7.62 | 0.30 |
| **31** | BAK00297.1 | predicted protein [Hordeum vulgare subsp. vulgare] | 71 | 0.45 | 47.230/8.96 | 0.46 |
| **37** | GAQ83961.1 | hypothetical protein KFL_001700160 [Klebsormidium nitens] | 75 | 0.59 | 29.101/9.55 | 0.29 |
| **40** | KCW60946.1 | hypothetical protein EUGRSUZ_H03679, partial [Eucalyptus grandis] | 76 | 0.35 | 133.442/8.41 | 0.38 |
| **69** | XP_022007088.1 | uncharacterized protein LOC110906091 [Helianthus annuus] | 84 | 0.47 | 22.443/6.90 | 0.92 |
| **98** | KXZ45947.1 | hypothetical protein GPECTOR_49g531 [Gonium pectorale] | 74 | 0.93 | 11.939/9.81 | 0.41 |
| **101** | AIA25753.1 | hypothetical protein, partial [Phyllostachys nigra var. henonis] | 66 | 0.69 | 32.784/8.53 | 0.34 |
| **108** | OAE25752.1 | hypothetical protein AXG93_4368s2120 [Marchantia polymorpha subsp. ruderalis] | 71 | 0.62 | 46.123/9.04 | 0.49 |
| **109** | XP_009361749.1 | PREDICTED: inorganic pyrophosphatase 2-like [Pyrus x bretschneideri] | 70 | 0.47 | 32.351/6.36 | 0.47 |
| **112** | AML78684.1 | putative LOV domain-containing protein [Melissa officinalis] | 76 | 0.24 | 114.120/8.20 | 0.48 |
| **114** | OMO84676.1 | hypothetical protein COLO4_21905 [Corchorus olitorius] | 76 | 0.48 | 62.925/9.00 | 0.48 |
| **115** | XP_021774837.1 | uncharacterized protein LOC110738730 isoform X1 [Chenopodium quinoa] | 72 | 0.34 | 53.505/9.02 | 0.27 |
| **116** | ACU20174.1 | unknown [Glycine max] | 66 | 0.42 | 56.636/7.66 | 0.46 |
| **117** | GAU34576.1 | hypothetical protein TSUD_29220 [Trifolium subterraneum] | 63 | 0.38 | 36.241/7.57 | 0.34 |
| **118** | XP_020193248.1 | atherin-like [Aegilops tauschii subsp. tauschii] | 63 | 0.42 | 12.418/12.48 | 0.35 |
| **120** | XP_008783514.1 | PREDICTED: SAP-like protein BP-73 isoform X3 [Phoenix dactylifera] | 70 | 0.37 | 29.875/9.75 | 0.30 |
| **121** | XP_001759565.1 | predicted protein [Physcomitrella patens] | 65 | 0.51 | 111.876/9.16 | 0.34 |
| **123** | AFK42845.1 | unknown [Lotus japonicus] | 73 | 0.69 | 11.735/6.73 | 0.41 |
| **124** | EMS53676.1 | hypothetical protein TRIUR3_31327 [Triticum urartu] | 69 | 0.64 | 14.749/5.90 | 0.32 |
| **127** | XP_021759626.1 | uncharacterized protein LOC110724512 [Chenopodium quinoa] | 69 | 0.37 | 46.371/9.50 | 0.15 |

The spot number corresponds to the number shown in Figure S6. MS, mascot score; SC, sequences coverage; MW, molecular weight; pI, isoelectric points; FC, fold change.

# Supplementary Table S11. Comparison of the transcriptomic effects of different beneﬁcial and pathogenic fungi on their hosts.

| **Fungus type** | **Fungus-Host plant** | **Analysis** | **DEG*** | **Trend** | **Up-regulated genes or pathway** | **Down-regulated genes or pathway** | **References** |
| --- | --- | --- | --- | --- | --- | --- | --- |
| **Beneficial fungus/ endophyte** | *Gilmaniella* sp. AL12 -*Atractylodes lancea* | RNA-seq | 2.7% (4019) | None | Primary metabolism, Secondary metabolism | Hormone signaling, plant-pathogen interaction | This study |
|  | *Trichoderma harzianum* -*Arabidopsis thaliana* | Microarray | 0.3% (66) | None | Regulation of transcription | Plant stress response | Morán-Diez et al, 2012 |
|  | *Trichoderma harzianum* - *Vitis vinifera* | RNA-seq | 1% (7024) | Up | Microbial recognition, Defence-related processes | Disease-related processes | Perazzolli et al., 2012 |
|  | *Epichloe coenophiala*- *Lolium arundinaceum* | RNA-seq | 0.51% (478) | None | Ribosomal proteins | Response to chitin, ROS burst during defence response, intracellular signal transduction | Dinkins et al., 2017 |
|  | *Metarhizium anisopliae Arachis hypogaea* | RNA-seq | 0.2% (164) | Down | oxidation-reduction process, transport process, metabolic process | hypersensitive response (HR), defence response | Hao et al., 2017 |
|  | *Piriformospora indica-Glycine max* | RNA-seq | 0.66% (299) | Down | Anabolic processes | Biotic Stress Response, Catabolic processes | Bajaj et al., 2018 |
| **Beneficial fungus/ mycorrhiza** | *Rhizophagus irregularis* -*Medicago truncatula* | Microarray | 0.5% (297) | Up | Secondary and hormone metabolism, gene expression and RNA metabolism, primary metabolism, defence and cell rescue | Abiotic stimuli and development, signal transduction and post-translational regulation, etc | Adolfsson et al., |
|  | *Rhizophagus irregularis -Poncirus trifoliata* | RNA-seq | 5.5% (909) | Up | Phosphorus, sugar and plant hormone metabolism |  | Chen et al., 2017 |
|  | *Funneliformis mosseae* - *Solanum lycopersicum* | RNA-seq | 3.3% (712) | Up | Photosynthesis, stress response, transport, amino acid synthesis and carbohydrate metabolism | Cell wall, metabolism and ethylene response pathways. | Zouari et al., 2014 |
| **Pathogen/**  **necrotroph** | *Botrytis cinereal -*  *Lactuca sativa* | RNA-seq | 23.4% (4598) | Up | Phenylpropanoid pathway,  Terpenoid biosynthesis | Photosynthesis | De Cremer et al., 2013 |
| **Pathogen/**  **biotroph** | *Ustilago maydis-Zea mays* | Microarray | 22% (2420) | Up | Hormone signaling, Antioxidant and secondary metabolism, glycolysis and the TCA cycle | Defence response, Photosynthesis | Doehlemann et al., 2008 |
| **Pathogen/**  **hemibiotroph** | *Magnaporthe oryzae-Oryza sativa* | RNA-seq | 13.9% (16048) | Up | Pathogenesis-related and phytoalexin biosynthetic genes |  | Kawahara et al., 2012 |


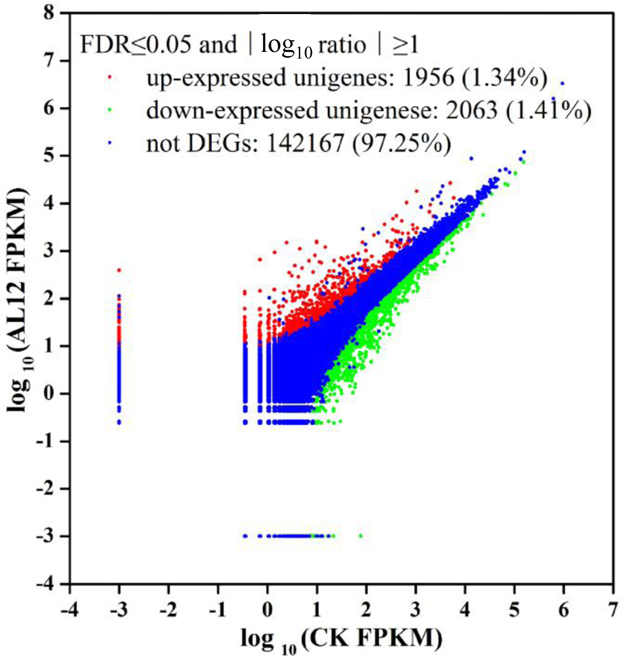


# Supplementary Figure S1. Scatter-plot graph of DEGs pattern from transcriptome of *A. lancea* after *Gilmaniella* sp. AL12 inoculation.


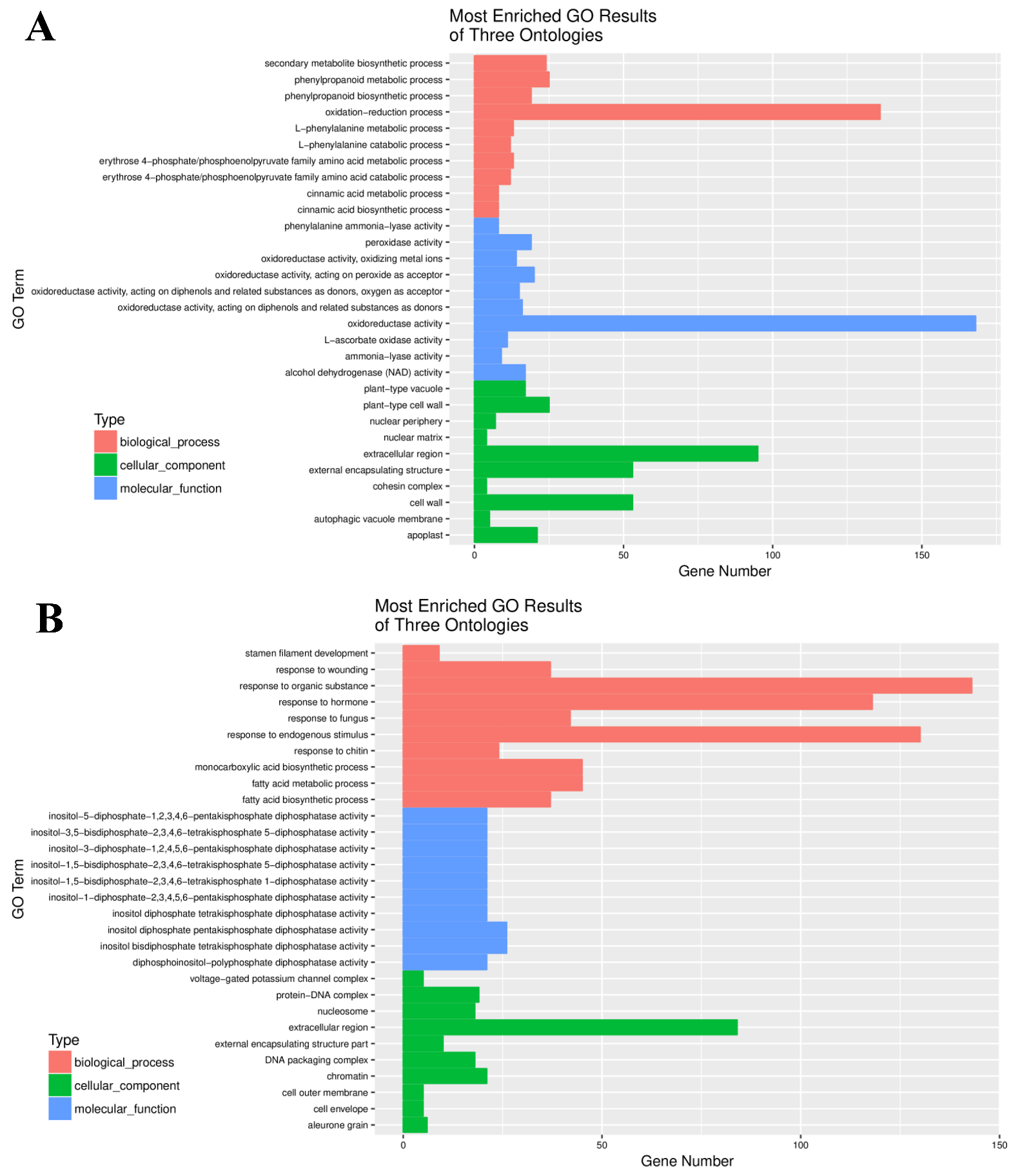


# Supplementary Figure S2. Most enriched GO results related to biological process, cellular component, and molecular function among AL12 upregulated (A) and AL12 downregulated genes (B). The y axis indicates the GO terms. The x axis presents the number of DEGs.

# Supplementary Figure S3.
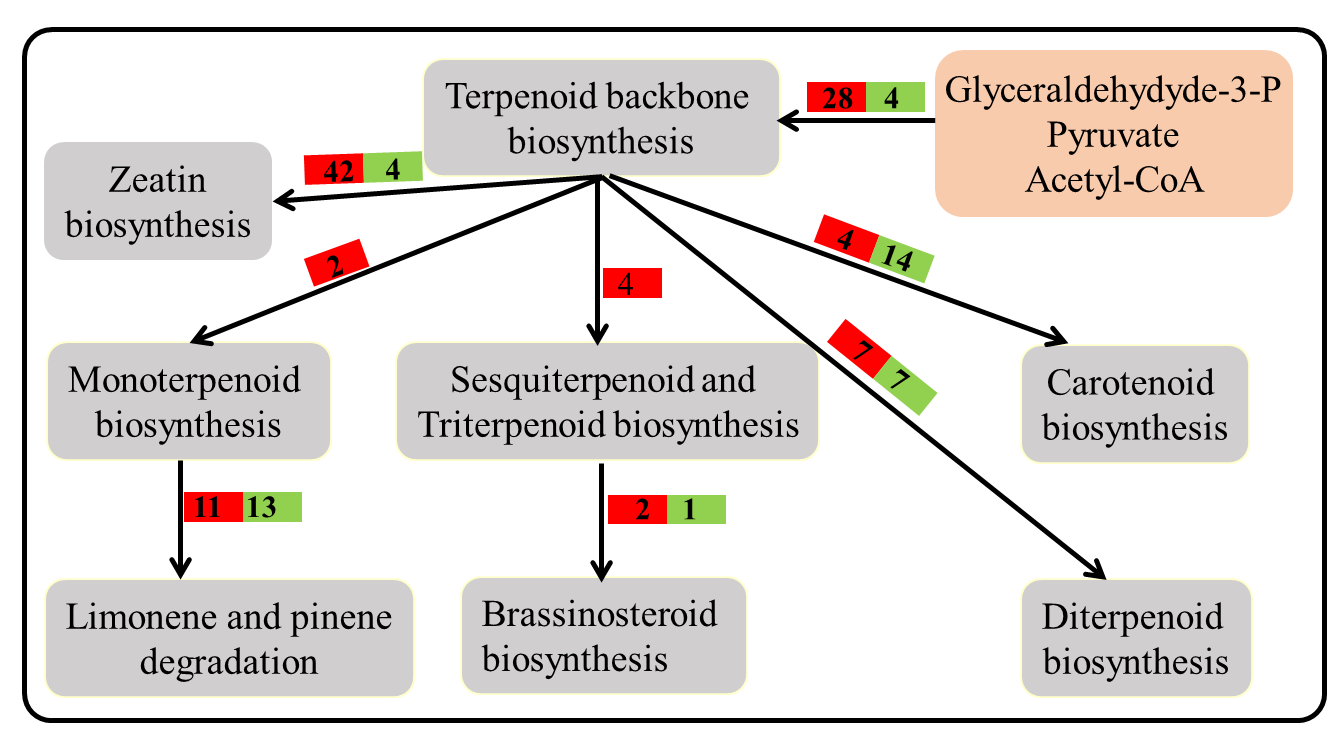
 Distribution of DEGs involved in the terpenoid metabolism pathway. The grey block indicates the KEGG pathway. The pink block represents terpenoid precursor substances. The numbers in red or green blocks represent the number of up or downregulated DEGs, respectively. Glyceraldehydyde-3-P, glycerate-3-phosphate.


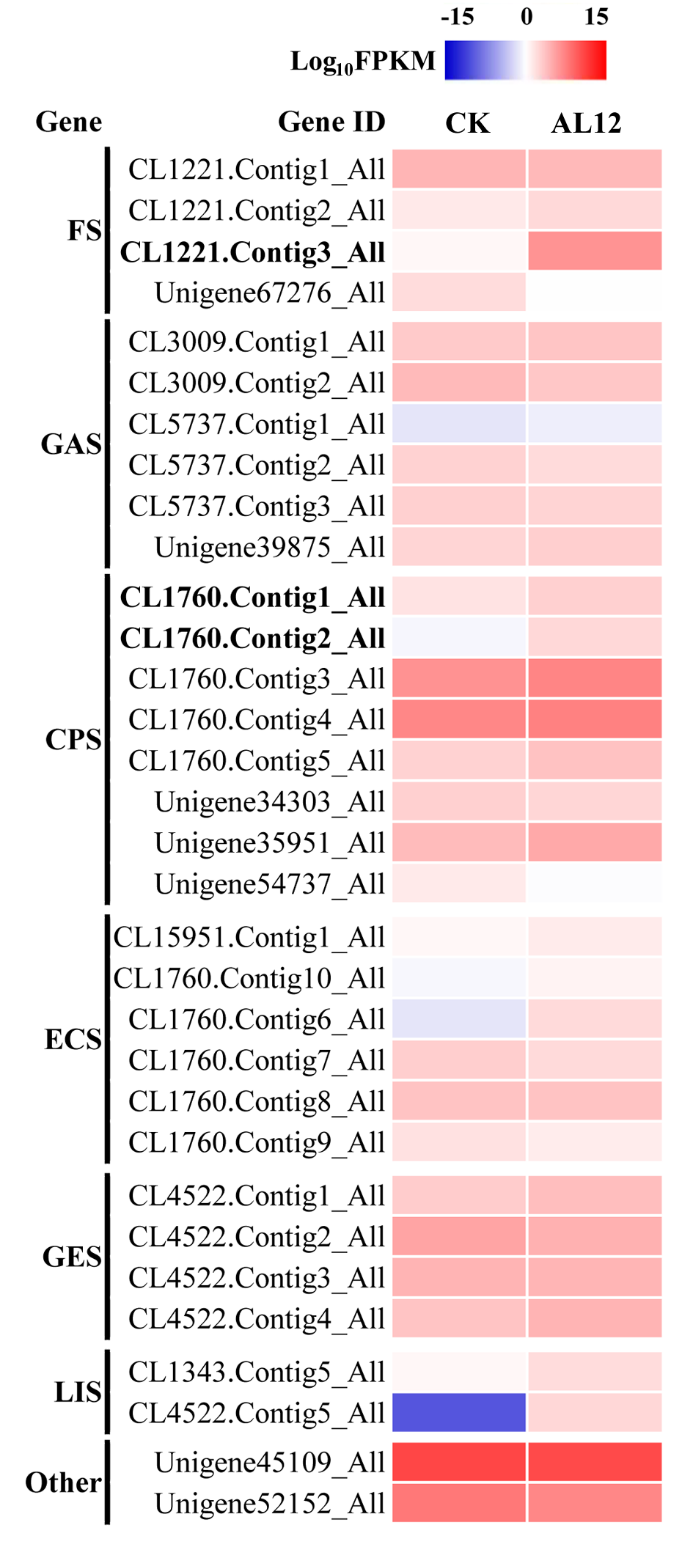


# Supplementary Figure S4. Expression of DEGs annotated as terpene synthase following AL12 inoculation. FS, β-farnesene synthase; GAS, germacrene A synthase; CPS, β-caryophyllene synthase; ECS, epi-cedrol synthase, GES, geraniol synthase, LIS, R-linalool synthase.

#

Supplementary Figure S5. Distribution of DEGs annotated as transcription factors under AL12 inoculation.

#
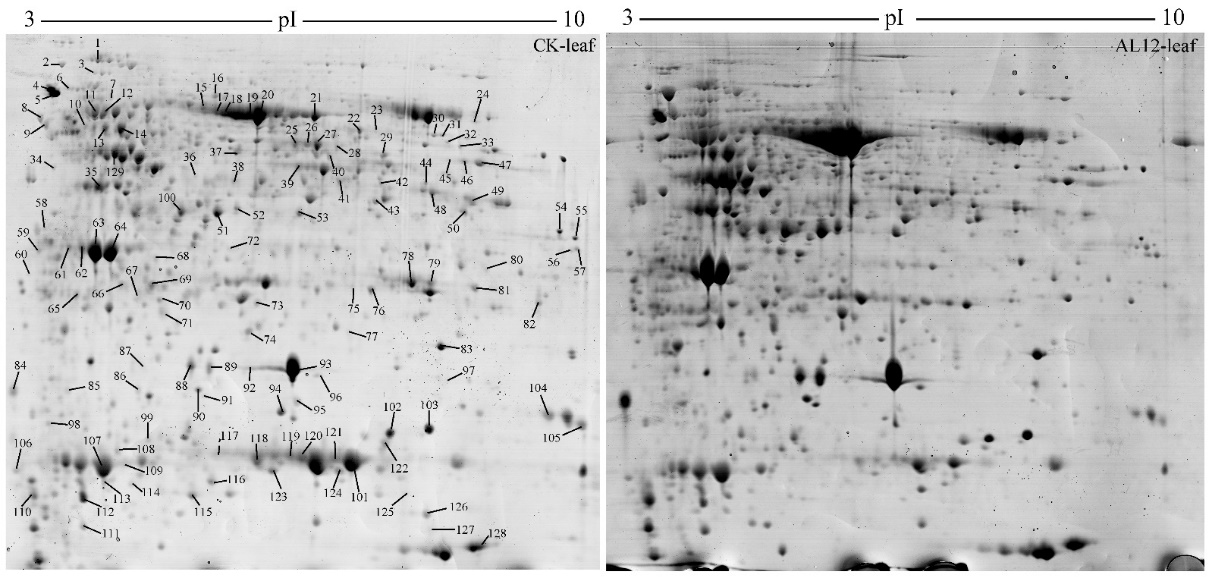
Supplementary Figure S6. Representative 2-DE gels of shoots proteins extracted from endophyte-inoculated and free-inoculated *A. lancea*. Differentially regulated proteins are indicated by arrows and numbered from 1 to 129.

#

Supplementary Figure S7. Functional classification of differentially expressed proteins in *A. lancea* after AL12 inoculation. The x axis indicates protein categories. The y axis indicates the number of differentially expressed proteins.

**
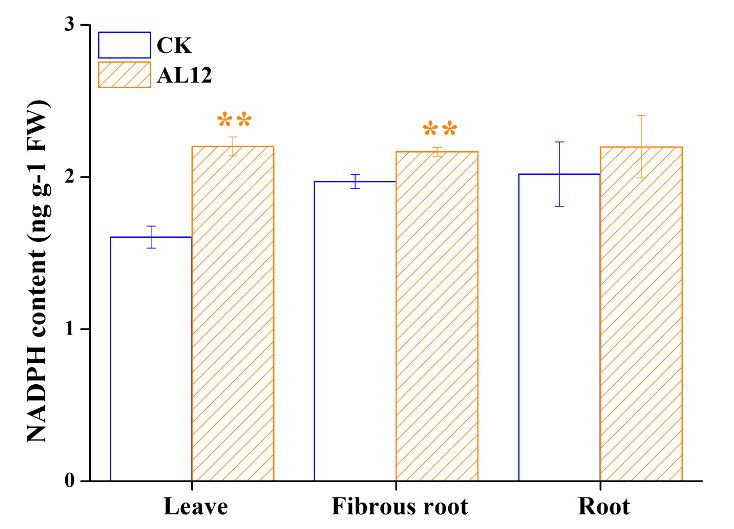
**

**Supplementary Figure S8.** The effect of the fungal endophyte *Gilmaniella* sp. AL12 inoculation on the content of NADPH in shoots of *A. lancea*. Thirty-day-old plantlets treated with 5-mm AL12 mycelial disks were harvested at 15 DPI. Controls were established using equal sized potato dextrose agar disks. Values are the means of three independent experiments. Bars represent standard deviations. Asterisks denote signiﬁcant differences from the control (*t*-test; **P* < 0.05; ***P* < 0.01).

**Supplementary Materials and Methods**

# Quantification of NADPH

Thirty-day-old plantlets treated with 5-mm AL12 mycelial disks or potato dextrose agar disks were harvested at 15 DPI. Shoot tissue of endophyte-inoculated or endophyte-free plants were harvested to determine the content of NADPH. Fresh samples (0.5 g) were ground with 5 ml 0.1 M phosphate buffer solution (pH 7.4), and then centrifuged (3000 g, 20 min, 4 °C). The supernatant was used for NADPH measurement. The content of NADPH was measured using the Plant NADPH ELISA Kit (Shanghai Enzyme-linked Biotechnology Co., China) following the manufacturer’s instructions. The standard curve was Y = (X-0.1242)/0.003*0.005*4, R^2^= 0.996.
